# Optimized fertilizer–microbe ratios enhance synergistic restoration of alpine mining ecosystems

**DOI:** 10.3389/fmicb.2025.1709528

**Published:** 2025-12-12

**Authors:** Zongcheng Cai, Jianjun Shi, Shouquan Fu, Fayi Li, Liangyu Lv, Qingqing Liu, Hairong Zhang, Shancun Bao

**Affiliations:** 1 Academy of Animal Husbandry and Veterinary Sciences, Qinghai University, Xining, China; 2 Key Laboratory of Adaptive Management of Alpine Grassland, Xining, China; 3 State Key Laboratory of Ecology and Plateau Agriculture and Animal Husbandry in Sanjiangyuan Jointly Established by the Ministry of Provincial Affairs, Qinghai University, Xining, China

**Keywords:** Muli coal mine, Qinghai–Tibet plateau, microbial inoculant, forage-specific fertilizer, bacterial community structure, dose–response relationship, ecological restoration

## Abstract

**Introduction:**

To address the critical issues of soil fertility depletion, poor vegetation establishment, and functional degradation of microbial communities in degraded alpine mining ecosystems, this study focuses on the Muli coal mine on the Qinghai–Tibet Plateau. This study systematically examined the synergistic regulatory mechanisms underlying the combined application of compound microbial inoculants and forage-specific fertilizers for restoring degraded ecosystems. The findings establish a theoretical framework and technical approach for rehabilitating fragile alpine ecosystems under extreme environmental conditions.

**Methods:**

A three-year field experiment was conducted using a long-term plot design. Integrated assessments of vegetation performance, soil physicochemical properties, and soil bacterial community structure (via high-throughput 16S rRNA gene sequencing) were performed. Multivariate statistical analyses, including redundancy analysis (RDA) and structural equation modeling (SEM), were employed to evaluate the comprehensive effects of fertilizer–microbe co-application on the vegetation–soil–microorganism system.

**Results and discussion:**

The optimized fertilizer–microbe treatment (W3J1: 375.00 kg·hm^−2^ forage-specific fertilizer + 350.00 kg·hm^−2^ compound microbial inoculant) significantly promoted vegetation growth, increased soil carbon and nitrogen contents, and effectively alleviated soil salinization. This treatment reshaped soil bacterial community structure, enriched functional taxa (e.g., *Pseudomonadota* and *Bacteroidota*), and enhanced the complexity and functional potential of microbial interaction networks. Mechanistic analysis revealed that the synergistic effect of fertilizer and microbes primarily drove ecosystem recovery through a dual-pathway mechanism: direct microbial regulation and vegetation–soil feedback. The direct effect on bacterial *α*-diversity was extremely significant (*β* = 0.76, *p* < 0.001). Soil pH was identified as the key driver of microbial community assembly, while excessive fertilization induced salinity rebound, indicating a clear application threshold. This study provides a theoretical basis and technical support for the precise ecological restoration of fragile ecosystems in alpine mining areas.

## 1 Introduction

Alpine mining ecosystems, due to their unique geographical settings and fragile ecological balance, often face severe challenges in ecological restoration following anthropogenic mining disturbances (Liu et al., 2024). Typical alpine mining areas, such as the Muli mining region on the Qinghai–Tibet Plateau, must not only address common issues like soil and water loss prevalent in conventional mining sites, but also overcome multiple limitations imposed by extreme environmental conditions, such as low temperature and hypoxia, that restrict plant growth and microbial activity (Lyu et al., 2024). In recent years, with the rapid advancement of microbial ecological theory, ecological restoration technologies based on vegetation–soil–microorganism interaction mechanisms have become a key breakthrough for addressing the persistent challenges of ecosystem recovery in alpine mining environments.

A growing body of evidence indicates that microbial inoculants can significantly enhance the ecological functions of degraded mine soils through three synergistic mechanisms: promoting soil nutrient transformation, activating rhizosphere microbial activity, and reconstructing soil microbial community structure (Jia et al., 2024; Ren et al., 2024). As core functional units of soil ecosystems, microbial communities play a pivotal role in driving soil structural improvement and functional recovery (Attoye et al., 2025). For instance, a study on mine tailings in the Arctic Svalbard archipelago demonstrated that functional microbes enhance aggregate stability in cold-region soils by up to 47% through nitrogen and phosphorus mobilization and extracellular polymeric substance (EPS) secretion, and that the establishment of symbiotic networks between these microbes and pioneer plants serves as a key driver of ecosystem restoration (Erikstad et al., 2023). In a semi-arid mining area in northwestern China, Jiao et al. (2023) reported that inoculation with arbuscular mycorrhizal fungi (AMF) significantly increased the activities of soil invertase, catalase, urease, and alkaline phosphatase by 23.8, 21.3, 18.8, and 8.6%, respectively (*p* < 0.05). Qiu et al. (2019) further confirmed that AMF inoculation significantly improves soil nutrient availability and enzymatic activity, thereby promoting plant growth in nutrient-poor soils. Field experiments by Oliveira et al. (2022) showed that the application of compound microbial inoculants significantly enhances plant productivity, increasing soybean yield by 20–35%. Kang et al. (2016) isolated highly tolerant indigenous rhizobia from a vanadium-titanium magnetite mine wasteland, which were capable of forming effective symbioses with leguminous crops under high heavy-metal stress, providing valuable microbial resources for bioremediation in alpine mining environments.

Traditional ecological restoration practices in mining areas have primarily relied on physical engineering measures and simplistic vegetation reestablishment techniques, with insufficient understanding of the regulatory role of soil microbial communities in ecosystem functional recovery (Qiao et al., 2025). Emerging evidence indicates a significant correlation between the successional dynamics of soil microbial community structure and ecosystem functional restoration in alpine mining regions, where changes in the abundance of key functional microbial taxa, such as *Actinobacteria* and *Proteobacteria*, directly influence soil nutrient cycling efficiency and primary productivity (Peng et al., 2025). In a study conducted in arid mining areas, Tu (2015) demonstrated that the application of a compound microbial inoculant increased soil moisture content by 18.7%, reduced bulk density by 12.3%, and enhanced available nitrogen and phosphorus concentrations by 32.5 and 28.9%, respectively. Soil bacterial abundance reached 3.6 × 10^7^ CFU·g^−1^, while actinomycete abundance reached 4 × 10^6^ CFU·g^−1^. Xiao et al. (2021), through metagenomic analysis, found that the relative abundance of nitrogen-fixing bacteria in the microbial inoculant treatment was 2.8-fold higher than in the control, and the expression levels of genes associated with carbon cycling were increased by 1.5–2.3-fold. A long-term field experiment by Yuan et al. (2013) further confirmed that microbial inoculant application enhanced the soil organic carbon accumulation rate by 25.6% in bauxite mine restoration areas, with restoration efficacy showing a significant increasing trend over application years. However, systematic studies on the synergistic effects of compound microbial inoculants and chemical fertilizers in alpine mining ecosystems remain limited, particularly with regard to the quantitative understanding of microbial–nutrient interactions, which requires urgent advancement (Liu T. N. et al., 2025; Liu M. et al., 2025; Liu H. H. et al., 2025; Liu Q. Q. et al., 2025).

Forage-specific fertilizer, as a blended fertilizer, has become the preferred chemical fertilizer in vegetation restoration projects in alpine mining areas due to its highly adjustable nutrient ratios and flexible application methods (Meng et al., 2025). However, conventional fertilization practices often overlook the potential inhibitory effects of chemical fertilizers on soil microbial communities (Zhang et al., 2024). Current evidence indicates that the co-application of microbial inoculants with chemical fertilizers at appropriate rates can effectively mitigate the negative impacts of fertilizers on soil micro-ecosystems. Fu et al. (2023) demonstrated that combined application of a compound microbial inoculant with chemical fertilizer, using a 50% nitrogen fertilizer substitution rate, significantly improved soil physical structure, increasing soil porosity and moisture content by 12.03 and 18.34%, respectively. A field experiment by Wang et al. (2014) on an open-pit mine spoil heap further showed that co-application of a compound microbial inoculant with blended chemical fertilizer increased soil available nitrogen and phosphorus by 130 and 217%, respectively. Concurrently, soil microbial abundance was significantly enhanced, with bacterial, actinomycete, and fungal populations increasing by 103, 68, and 20%, respectively, while growth parameters of *Medicago sativa* (alfalfa) were markedly improved. These findings provide critical theoretical support and practical guidance for the application of the combined microbial inoculant and forage-specific fertilizer strategies in the ecological restoration of alpine mining ecosystems.

Based on the aforementioned research background, this study proposes the following core scientific hypotheses: (1) Combined application of a compound microbial inoculant and forage-specific fertilizer can significantly improve the micro-environmental conditions of degraded alpine mining soils by modulating the community structure and metabolic activity of key functional microbial taxa; (2) a dose-dependent response exists in the co-application, with potential inhibitory effects occurring when the optimal ratio threshold is exceeded. To test these hypotheses, a three-year field-based controlled experiment was conducted using constructed grasslands in the Muli mining area on the Qinghai–Tibet Plateau as the study system. The mechanisms by which different co-application regimes influence vegetation community characteristics, soil physicochemical properties, and microbial community assembly were systematically investigated. Particular emphasis was placed on: (1) Characteristics of soil bacterial diversity and evolutionary patterns of co-occurrence network topology across different fertilizer–inoculant ratios; (2) interaction networks between key functional microbial taxa and environmental factors, along with associated ecological functions.

The findings not only provide a theoretical basis for microbe–nutrient synergistic regulation in the restoration of degraded ecosystems in alpine mining regions, but also offer practical guidance for optimizing ecological restoration technologies in the Qinghai–Tibet Plateau mining areas. This study holds significant scientific value and practical implications for strengthening regional ecological security barriers and promoting sustainable development.

## 2 Materials and methods

### 2.1 Study site description

The study site was located in the coal reserve area of Muli Town, Tianjun County, Qinghai Province, China (38°9′34″N, 99°9′40″E), at an elevation of approximately 4,200 m above sea level, with geographical location shown in Supplementary Figure S1a. The region was characterized by a typical alpine subarctic semi-arid steppe climate, exhibiting pronounced high-altitude cold conditions, including a long winter lasting 7–8 months, a short summer, and large diurnal temperature fluctuations. Precipitation was seasonally distributed, with the rainy season occurring from June to August, while snowfall dominated from November to May of the following year. Climatic observations indicated that the site had a mean annual sunshine duration of 2164.9 h, a mean annual air temperature of −5.3 °C, a mean annual precipitation of approximately 626 mm, and a mean annual evaporation rate of 1,049.9 mm. Solar radiation was intense, with annual sunshine duration ranging from 2,551 to 3,332 h (Liu et al., 2024). Due to the harsh climatic conditions, the vegetation growing season was limited to approximately 120 days. Detailed climatic characteristics were presented in Supplementary Figure S1b.

The surface matrix of the degraded grassland at the study site was primarily composed of coal gangue and rock debris. Soil physicochemical analysis revealed the following properties: total nitrogen content of 1.05 g·kg^−1^, total phosphorus content of 0.84 g·kg^−1^, organic matter content of 60.34 g·kg^−1^, electrical conductivity of 19,090 μS·cm^−1^, pH value of 8.50, and moisture content of 15.0%. The original vegetation type was a typical alpine meadow, with dominant species including *Carex parvula* O. Yano, *Carex alatauensis* S. R. Zhang, and *Elymus nutans* Griseb. (Liu T. N. et al., 2025; Liu M. et al., 2025; Liu H. H. et al., 2025; Liu Q. Q. et al., 2025). Field conditions of the study area are shown in Supplementary Figure S2.

### 2.2 Experimental materials

The compound microbial inoculant used in this study is a water-soluble powder developed by Hubei Qiming Bioengineering Co., Ltd. (Wuhan, China), designated as “Compound Microbial Inoculant” for research purposes. It has a viable microbial concentration of no less than 1 × 10^8^ CFU·g^−1^ and was rationally formulated based on functional synergy among key microbial strains. The functional core of the inoculant consists exclusively of three well-defined strains: *Trichoderma harzianum* (strain THZ-1), *Bacillus subtilis* (strain BS-15), and *Streptomyces albus* (used as a representative actinomycete), combined at a mass ratio of 2:1:1. These three strains constitute the sole intentionally added microbial components; no other microbial species or strains were included in the formulation. The carrier system comprises maltodextrin and modified cellulose, supplemented with trace nutrients including yeast extract and KH₂PO₄ to support microbial viability during storage and early soil colonization. Notably, the formulation contains no synthetic preservatives or chemical stabilizers, ensuring environmental compatibility and safety.

It should be noted that this inoculant is currently at the stage of field evaluation and formulation optimization and has not yet entered large-scale production or commercial distribution. Therefore, it does not carry an official product registration number. Should this formulation be commercialized in the future, full product information will be disclosed to ensure research traceability and reproducibility. Quality control analyses confirmed low levels of heavy metals: cadmium (Cd) 0.32 mg·kg^−1^, chromium (Cr) 5.18 mg·kg^−1^, and arsenic (As) 0.63 mg·kg^−1^, all substantially below the limits specified in the Chinese National Standard for Agricultural Microbial Inoculants (GB 20287–2006; Cd ≤ 10 mg·kg^−1^, Cr ≤ 100 mg·kg^−1^, As ≤ 10 mg·kg^−1^). Fecal coliforms were below the detection limit (<100 MPN·g^−1^), indicating compliance with hygienic safety requirements for microbial agricultural products.

The forage-specific blended fertilizer (N–P₂O₅–K₂O = 18–12–5) used in the experiment was produced by Gansu Sudi Fertilizer Co., Ltd. and strictly conformed to the *Blended Fertilizers (BB Fertilizers)* standard (GB/T 21633–2020). The total nutrient content (N + P₂O₅ + K₂O) was ≥35.00% by mass, meeting the national regulatory requirements. Analytical testing confirmed the nitrogen (N), phosphorus (P₂O₅), and potassium (K₂O) contents to be 18.00, 12.00, and 5.00%, respectively. The nutrient ratios were scientifically balanced and sufficient to meet the nutritional requirements of forage grasses in alpine mining environments.

Four cold-tolerant grass species were selected for the experiment: *Festuca sinensis* Keng, *Poa pratensis* L., *Poa crymophila* Keng, and *Elymus breviaristatus* Keng f. All seed materials were provided by the Institute of Grassland Science, Academy of Animal and Veterinary Sciences, Qinghai University. Seed purity was ≥92%, and germination rate was ≥85%. These grass species exhibit excellent adaptability to alpine environments.

### 2.3 Experimental design

#### 2.3.1 Design of the experiment

A randomized complete block design was employed in this study. The experiment included 10 treatment combinations: nine co-application treatments (3 levels of microbial inoculant × 3 levels of fertilizer) and one control (CK). Each treatment was replicated three times, resulting in a total of 30 experimental plots. Based on previous research, soil fertility measurements, and nutrient requirements of constructed grasslands (Wang et al., 2022), the following fertilization gradients were established: forage-specific fertilizer application rates of 225.00, 300.00, and 375.00 kg·hm^−2^, and compound microbial inoculant application rates of 350.00, 500.00, and 650.00 kg·hm^−2^. Specific treatment combinations were detailed in Table 1. Fertilization was conducted in early June of 2023 and 2024. The materials were applied manually and immediately incorporated into the soil via light raking.

**Table 1:** Experimental design for different fertilization treatments.

Treatment group	Forage-specific fertilizer (kg·hm^−2^)	Compound microbial inoculant (kg·hm^−2^)
CK	—	—
W1J1	225.00	350.00
W1J2	225.00	500.00
W1J3	225.00	650.00
W2J1	300.00	350.00
W2J2	300.00	500.00
W2J3	300.00	650.00
W3J1	375.00	350.00
W3J2	375.00	500.00
W3J3	375.00	650.00

W denotes the application rate of forage-specific fertilizer (W1: 225.00 kg·hm^−2^, W2: 300.00 kg·hm^−2^, W3: 375.00 kg·hm^−2^), and J denotes the application rate of compound microbial inoculant (J1: 350.00 kg·hm^−2^, J2: 500.00 kg·hm^−2^, J3: 650.00 kg·hm^−2^).

The control group (CK) represented a basic restoration approach without nutrient or microbial amendments. CK plots underwent the same land preparation and vegetation establishment procedures as the other treatment groups, including soil tilling, leveling, and sowing of the same cold-tolerant grass species, but did not receive applications of grass-specific fertilizer or composite microbial inoculant. This experimental design allowed for the assessment of natural recovery processes driven solely by artificial vegetation establishment and provided a baseline against which the additive effects of fertilization and microbial inoculation could be evaluated.

#### 2.3.2 Seeding and management

Vegetation establishment was carried out on May 5, 2022, using mechanical row seeding. The experimental materials consisted of four cold-tolerant grass species: *Festuca sinensis* Keng, *Poa pratensis* L., *Poa crymophila* Keng, and *Elymus breviaristatus* Keng f. The seeds were sown in equal proportions (1,1,1,1) with a seeding rate of 18 g·m^−2^ and a sowing depth of 2–3 cm. Immediately after sowing, a polypropylene non-woven fabric (72% ± 3% light transmittance, air permeability of 230 ± 15 L·m^−2^·s^−1^) was applied at a rate of 30 g·m^−2^ and secured using a cross-shaped fixation method with U-shaped soil nails installed at 2 m intervals. The mulch was removed gradually in three stages (at 5-day intervals) when seedlings reached a height of 5 cm.

#### 2.3.3 Field layout

Each experimental plot measured 20 m^2^ (4 m × 5 m), with 2 m-wide buffer strips between plots. A border row was established around the perimeter of the experimental area, and all treatments were arranged randomly. To minimize edge effects, a 0.5 m margin around the perimeter of each plot was excluded from sampling and observation.

### 2.4 Plant community survey and soil sample collection

Vegetation surveys were conducted in September 2023 and during the same period in 2024, corresponding to the end of the growing season. Measured parameters included key ecological indicators such as community height, canopy cover, density, and aboveground biomass. Detailed measurement data are presented in Table 2. To ensure representativeness and scientific rigor, five randomly selected 50 cm × 50 cm quadrats were established within each treatment plot. Quadrat placement followed these criteria (Lyu et al., 2024): (1) at least 50 cm from plot boundaries; (2) center-to-center distance between adjacent quadrats ≥1 m; (3) avoidance of areas with obvious topographic anomalies.

**Table 2:** Changes in vegetation height, cover, density, and aboveground biomass under different fertilization treatments.

Year	Treatment	Vegetation height (cm)	Vegetation coverage (%)	Vegetation density (plants·m^−2^)	Aboveground biomass (g·m^−1^)
2023	CK	16.53 ± 0.93 d	58.81 ± 1.42 d	345.33 ± 8.35 c	198.33 ± 8.19 c
	W1J1	21.11 ± 0.70 c	63.47 ± 0.98 c	385.67 ± 7.36 b	223.33 ± 8.17 c
	W1J2	26.31 ± 1.10 b	64.98 ± 1.45 c	408.67 ± 8.65 ab	266.33 ± 12.17 b
	W1J3	34.29 ± 0.63 a	75.32 ± 2.54 a	424.00 ± 11.33 a	325.00 ± 21.08 a
	W2J1	31.51 ± 2.25 a	69.67 ± 1.47 b	413.67 ± 10.99 a	315.33 ± 11.05 a
	W2J2	32.03 ± 1.45 a	71.02 ± 1.63 ab	420.67 ± 5.24 a	322.33 ± 12.86 a
	W2J3	32.74 ± 1.40 a	72.51 ± 1.94 ab	414.00 ± 4.04 a	339.67 ± 9.61 a
	W3J1	34.92 ± 1.68 a	74.31 ± 0.61 a	428.00 ± 9.07 a	342.67 ± 11.89 a
	W3J2	31.68 ± 0.78 a	72.49 ± 1.77 ab	411.33 ± 5.84 a	331.33 ± 3.76 a
	W3J3	31.72 ± 0.90 a	71.14 ± 0.92 ab	406.33 ± 13.38 a	326.67 ± 11.92 a
2024	CK	17.86 ± 1.06 e	59.14 ± 0.96 d	342.67 ± 10.48 c	195.67 ± 4.84 f
	W1J1	22.71 ± 0.75 d	65.13 ± 1.64 c	389.00 ± 4.04 b	233.33 ± 1.86 e
	W1J2	26.64 ± 1.15 c	68.98 ± 0.89 bc	422.00 ± 17.62 a	273.00 ± 6.08 d
	W1J3	35.16 ± 0.46 ab	76.98 ± 0.91a	440.33 ± 5.33 a	351.33 ± 7.22 a
	W2J1	32.18 ± 1.64 b	73.33 ± 2.03 ab	437.00 ± 14.36 a	321.00 ± 5.77 c
	W2J2	33.70 ± 0.67 ab	72.72 ± 2.30 ab	434.00 ± 12.5 a	329.00 ± 10.69 bc
	W2J3	34.07 ± 1.84 ab	73.84 ± 1.57 a	424.00 ± 8.14 a	339.67 ± 3.53 abc
	W3J1	35.87 ± 1.78 a	77.31 ± 1.13 a	434.67 ± 3.67 a	352.67 ± 4.98 a
	W3J2	33.01 ± 0.55 ab	73.49 ± 2.65 ab	421.33 ± 8.99 a	344.67 ± 10.37 ab
	W3J3	32.72 ± 0.61 ab	72.47 ± 1.27 ab	409.67 ± 13.38 a	323.33 ± 7.22 c

W denotes the application rate of forage-specific fertilizer (W1: 225.00 kg·hm^−2^, W2: 300.00 kg·hm^−2^, W3: 375.00 kg·hm^−2^), and J denotes the application rate of compound microbial inoculant (J1: 350.00 kg·hm^−2^, J2: 500.00 kg·hm^−2^, J3: 650.00 kg·hm^−2^). Data are presented as mean ± standard deviation (*n* = 3), with lowercase letters indicating significant differences among treatments at *P* < 0.05.

Plant height of the four grass species, *Festuca sinensis* Keng, *Poa pratensis* L., *Poa crymophila* Keng, and *Elymus breviaristatus* Keng f., was measured using a steel tape measure with 1 mm precision. Within each quadrat, 10 representative individuals of each species were randomly selected, and their natural height was recorded. The average of these measurements was used as the mean vegetation height for the quadrat. The total number of plants (N) within each quadrat was recorded to calculate vegetation density (D), according to the following equation (Lyu et al., 2024):


(1) $$D=\frac{N}{A},A=0.25\;m^{2}$$

To measure the canopy cover of plant communities, this study employed a modified point-intercept method. In contrast to the conventional point-intercept method, which records only the presence or absence of vegetation at a single point, the modified approach enhanced detection sensitivity for sparse vegetation and heterogeneous canopy structures by optimizing sampling grid density and contact criteria (Ruscalleda Alvarez et al., 2025). Briefly, a regular grid of 10 cm × 10 cm was established within each 50 cm × 50 cm quadrat, resulting in 100 observation points at the grid intersections. Using vertical projection, the number of points (n) at which the vegetation canopy intercepted the pin was recorded. Canopy cover (C, %) was calculated (see Equation 1).

For overlapping canopy layers, only one contact per point was counted to prevent overestimation of canopy cover (Ruscalleda Alvarez et al., 2025). Five replicate quadrats were established per plot, and the final canopy cover value was determined as the mean of the five replicate measurements. By standardizing grid layout and contact determination rules, this modified method improved measurement consistency and comparability under the patchy vegetation distributions typical of alpine mining areas.

Aboveground vegetation within each quadrat was harvested at ground level using ethanol-sterilized stainless-steel scissors. Fresh weight was recorded immediately, followed by oven drying: samples were first subjected to 105 °C for 30 min to halt enzymatic activity, then dried at 65 °C for 48 h until constant weight (defined as <0.01 g difference between two consecutive weighings). Dry biomass was determined using an analytical balance.

Soil samples from the 0–10 cm surface layer were collected simultaneously from each experimental plot using a five-point sampling method. A stainless-steel soil auger with a 5 cm internal diameter was used to collect cores following standard operating procedures (Xiao et al., 2025). After collection, samples were passed through a 2 mm sieve to remove visible roots and gravel. Soil from the same plot was thoroughly homogenized to prepare a composite sample, which was then divided into three subsamples for different treatments:

1. The first subsample was air-dried in the dark at 25 ± 2 °C for the determination of soil organic matter (SOM), total nitrogen (TN), and total phosphorus (TP);
2. The second subsample was immediately stored at −20 °C for the measurement of soil water content (SWC), pH, and soil electrical conductivity (SEC);
3. The third subsample was rapidly transferred to a −80 °C ultra-low-temperature freezer to preserve microbial community structure integrity and used for subsequent microbial community diversity sequencing analysis.

### 2.5 Measurement of indicators and analytical methods

#### 2.5.1 Soil physicochemical properties

All analyses were conducted according to the standardized procedures described in *Soil Agricultural Chemistry Analysis* (Third Edition) (Bao, 1999), with three independent replicates performed for each treatment. SEC was determined using a DDS-307A conductivity meter, with a 5:1 (v:w) water-to-soil ratio used to prepare the extraction solution. SWC was determined by drying samples at 105 °C to constant weight and calculating the mass loss. Soil pH was measured using a FE28 pH meter in a 2.5:1 (v:w) water-to-soil suspension. SOM content was determined by the potassium dichromate oxidation method with external heating, followed by titration with ferrous sulfate. TN content was analyzed using a Kjeltec 8,400 automated Kjeldahl system: samples were digested with sulfuric acid and catalyst, then distilled and titrated. TP content was determined after digestion with a perchloric acid–sulfuric acid mixture, followed by colorimetric analysis using the molybdenum-antimony-ascorbic acid method at 700 nm on a UV-1800 UV–Vis spectrophotometer.

#### 2.5.2 Soil bacterial DNA extraction, PCR amplification, and sequencing

DNA extraction: DNA extraction was performed using the E. Z. N. A.^®^ Soil DNA Kit (Omega Bio-tek, United States) according to the manufacturer’s instructions. Each soil sample was extracted in triplicate to minimize batch effects during the extraction process. The integrity of the extracted DNA was verified by 1% agarose gel electrophoresis, and DNA concentration and purity (A260/A280 ratio) were determined using a NanoDrop 2000 spectrophotometer (Thermo Scientific, USA). The coefficient of variation (CV) of DNA concentrations across the three replicates was consistently <5%, indicating high reproducibility and reliability of the extraction process.

The PCR amplification and sequencing library preparation (Liu et al., 2024): High-quality DNA extracts were used as templates for PCR amplification of the V3–V4 hypervariable region of the 16S rRNA gene using barcoded, sequence-specific primers 338F (5′-ACTCCTACGGGAGGCAGCAG-3′) and 806R (5′-GGACTACHVGGGTWTCTAAT-3′). The 50 μL reaction mixture contained 4 μL of 5 × TransStart FastPfu Buffer, 2 μL of 2.5 mM dNTPs, 0.8 μL each of forward and reverse primers (5 μM), 0.4 μL of TransStart FastPfu DNA Polymerase, and 10 ng of template DNA. Thermal cycling conditions were as follows: initial denaturation at 95 °C for 3 min, followed by 27 cycles of 95 °C for 30 s, 55 °C for 30 s, and 72 °C for 30 s, with a final extension at 72 °C for 10 min, and hold at 4 °C. PCR products were separated by 2% agarose gel electrophoresis, purified using a DNA Gel Extraction Kit (PCR Clean-Up Kit, YuHua), and quantified using a Qubit 4.0 fluorometer (Thermo Fisher Scientific, United States).

Sequencing library construction was performed using the NEXTFLEX Rapid DNA-Seq Kit, which included adapter ligation, magnetic bead-based removal of adapter-dimer artifacts, PCR enrichment of the library, and magnetic bead purification. Final libraries were sequenced on an Illumina Nextseq 2000 platform (Majorbio Bio-Pharm Technology, China) using paired-end sequencing (2 × 150 bp).

High-throughput sequencing data analysis (Liu et al., 2024): Raw sequencing reads were processed using fastp software (v0.19.6) for quality control, including the removal of low-quality bases (Phred quality score <20), reads containing N bases, and sequences shorter than 50 bp. Paired-end reads were merged into full-length sequences using FLASH (v1.2.11) with a minimum overlap of 10 bp and a maximum mismatch rate of 0.2. Samples were demultiplexed based on barcode and primer sequences, allowing zero mismatches in barcodes and up to two mismatches in primers.

Quality-filtered sequences were clustered into operational taxonomic units (OTUs) at a 97% similarity threshold using UPARSE (v7.1), during which chimeric sequences were simultaneously identified and removed. Sequences originating from chloroplasts and mitochondria were also excluded from further analysis. To minimize bias caused by unequal sequencing depth, all samples were normalized to 20,000 sequences per sample, achieving a Good’s coverage of 99.09%. Taxonomic assignment was performed using the RDP Classifier (v2.11) against the SILVA 16S rRNA database (v138), with a confidence threshold of 70%. Community composition was summarized at each taxonomic level. Functional potential of the microbial communities was predicted using FAPROTAX (v1.2.11), which infers potential biogeochemical functions related to nitrogen cycling from taxonomic annotations based on 16S rRNA gene sequences.

### 2.6 Data analysis and visualization

All measured variables were determined in triplicate. Raw data were first standardized and preprocessed using Microsoft Excel 2019, then subjected to statistical analysis in SPSS 27.0. One-way analysis of variance (ANOVA) was conducted to test for significant differences among treatments in vegetation parameters, soil physicochemical properties, and microbial community structure (significance level *α* = 0.05). For variables showing significant differences (*p* < 0.05), *post hoc* comparisons were performed using Duncan’s multiple range test to identify specific pairwise differences.

Principal component analysis (PCA) was performed to extract eigenvalues and eigenvectors of the evaluated indicators. The number of principal components was determined based on a cumulative explained variance of ≥80%. Component scores and a comprehensive evaluation score were calculated, and the ranking of the comprehensive scores was visualized using Origin 2022.

Structural equation modeling (SEM) was conducted using the *lavaan* package (v0.6–16) in R. All input variables were *Z*-score standardized prior to analysis to eliminate scale effects. Model parameters were estimated using maximum likelihood (ML) estimation. Model fit was evaluated using Fisher’s C test in conjunction with Akaike’s Information Criterion (AIC) and Bayesian Information Criterion (BIC) to select the optimal model. Standardized path coefficients (β) and their corresponding *p*-values were used to quantify the strength and significance of direct effects among variables. Model visualization was performed using the *semPlot* package.

Microbial diversity analyses were conducted on the Majorbio Bio-Pharm Cloud Platform^1^. Alpha diversity indices, including Chao1 and Shannon, were calculated using mothur. Differences in alpha diversity between groups were assessed using the Wilcoxon rank-sum test. Beta diversity was characterized by principal coordinates analysis (PCoA) based on the Bray–Curtis distance matrix, and the significance of community structural differences among groups was evaluated using permutational multivariate analysis of variance (PERMANOVA). Linear discriminant analysis (LDA) effect size (LEfSe) analysis was applied to identify differentially abundant taxa (indicator species) from phylum to genus level, with an LDA score >2 and *p* < 0.05 set as thresholds.

## 3 Results and analysis

### 3.1 Effects of combined application of microbial inoculant and forage-specific fertilizer on soil physicochemical properties in artificial grassland

The co-application of microbial inoculant and forage-specific fertilizer significantly improved soil physicochemical properties (Table 3). After two consecutive years of observation, the W3J1 treatment exhibited the most favorable performance in soil moisture retention and nutrient accumulation. In 2023 (the second year after establishment), the SWC in the W3J1 treatment reached 26.28%, which was 81.24% higher than that of the CK and significantly different (*p* < 0.05). Concurrently, SEC decreased to 875.67 μS·cm^−1^, and soil pH declined to 7.77, both showing significant differences compared to the CK group (*p* < 0.05). Furthermore, this treatment markedly enhanced soil nutrient contents: SOM, TN, and TP reached 263.85, 7.29, and 2.78 g·kg^−1^, respectively, representing increases of 81.97, 54.45, and 109.02% relative to CK (*p* < 0.05).

**Table 3:** Changes in soil physicochemical properties under different fertilization treatments.

Year	Treatment	SWC (%)	SEC (μs·cm^−1^)	pH	SOM (g·kg^−1^)	TN (g·kg^−1^)	TP (g·kg^−1^)
2023	CK	14.50 ± 0.71 d	2110.00 ± 54.28 a	8.92 ± 0.02 a	145.00 ± 4.35 d	4.72 ± 0.06 e	1.33 ± 0.07 e
	W1J1	19.82 ± 0.95 c	1525.67 ± 65.58 b	8.53 ± 0.10 b	211.68 ± 11.72 c	5.94 ± 0.28 d	1.77 ± 0.07 d
	W1J2	22.63 ± 1.05 bc	1242.00 ± 69.16 c	8.33 ± 0.08 bc	231.73 ± 5.18 bc	6.26 ± 0.20 cd	1.93 ± 0.06 d
	W1J3	25.86 ± 1.79 a	974.67 ± 40.99 de	7.63 ± 0.19 f	254.48 ± 13.66 ab	7.12 ± 0.24 ab	2.21 ± 0.07 c
	W2J1	24.98 ± 1.01 ab	1009.33 ± 16.86 de	8.20 ± 0.08 c	256.98 ± 11.32 ab	6.69 ± 0.10 bc	2.32 ± 0.03 bc
	W2J2	24.79 ± 0.82 ab	980.67 ± 79.21 de	7.90 ± 0.03 de	244.78 ± 5.01 ab	7.08 ± 0.25 ab	2.52 ± 0.07 b
	W2J3	24.77 ± 0.67 ab	998.67 ± 52.98 de	8.08 ± 0.05 cd	255.70 ± 8.11 ab	7.11 ± 0.04 ab	2.55 ± 0.16 ab
	W3J1	26.28 ± 0.85 a	875.67 ± 19.62 e	7.77 ± 0.08 df	263.85 ± 6.18 a	7.29 ± 0.05 a	2.78 ± 0.09 a
	W3J2	24.75 ± 0.87 ab	1053.00 ± 98.80 cde	8.12 ± 0.06 cd	240.31 ± 9.72 ab	6.97 ± 0.03 ab	2.51 ± 0.08 b
	W3J3	24.64 ± 0.58 ab	1069.33 ± 90.89 cd	8.16 ± 0.09 cd	237.35 ± 6.09 bc	6.88 ± 0.05 ab	2.49 ± 0.04 b
2024	CK	15.83 ± 0.81 d	2076.67 ± 29.85 a	8.75 ± 0.09 a	136.66 ± 2.30 d	4.75 ± 0.08 e	1.23 ± 0.05 f
	W1J1	20.49 ± 0.56 c	1492.33 ± 43.21 b	8.50 ± 0.08 b	218.34 ± 11.78 c	6.10 ± 0.15 d	1.87 ± 0.07 e
	W1J2	22.63 ± 1.05 bc	1208.67 ± 55.32 c	8.23 ± 0.03 c	238.40 ± 10.11 bc	6.36 ± 0.22 cd	2.33 ± 0.20 d
	W1J3	26.86 ± 1.62 a	941.33 ± 20.34 de	7.57 ± 0.14 f	261.14 ± 8.82 ab	7.15 ± 0.22 ab	2.87 ± 0.06 a
	W2J1	26.31 ± 0.96 a	976.00 ± 16.52 de	8.13 ± 0.10 cd	260.31 ± 3.63 ab	6.76 ± 0.06 bc	2.36 ± 0.06 cd
	W2J2	25.79 ± 0.66 a	947.33 ± 75.11 de	7.84 ± 0.10 e	254.78 ± 2.02 ab	7.18 ± 0.26 ab	2.62 ± 0.06 abc
	W2J3	25.43 ± 1.14 ab	932.00 ± 15.14 de	8.04 ± 0.02 cde	256.37 ± 6.07 ab	7.27 ± 0.13 a	2.69 ± 0.12 ab
	W3J1	27.28 ± 1.56 a	855.67 ± 24.46 e	7.44 ± 0.05 f	267.18 ± 9.24 a	7.35 ± 0.08 a	2.85 ± 0.06 a
	W3J2	25.41 ± 0.52 ab	1019.67 ± 67.37 d	8.06 ± 0.03cde	243.65 ± 12.64 ab	7.00 ± 0.06 ab	2.61 ± 0.10 abcd
	W3J3	25.08 ± 0.54 ab	1036.00 ± 59.86 d	7.96 ± 0.05 de	247.35 ± 4.17 ab	6.94 ± 0.02 ab	2.53 ± 0.06 bcd

W denotes the application rate of forage-specific fertilizer (W1: 225.00 kg·hm^−2^, W2: 300.00 kg·hm^−2^, W3: 375.00 kg·hm^−2^), and J denotes the application rate of compound microbial inoculant (J1: 350.00 kg·hm^−2^, J2: 500.00 kg·hm^−2^, J3: 650.00 kg·hm^−2^). Data are presented as mean ± standard deviation (*n* = 3), with lowercase letters indicating significant differences among treatments at *P* < 0.05. SWC, soil water content; SEC, soil electrical conductivity; pH, soil pH; SOM, soil organic matter; TN, total nitrogen; TP, total phosphorus.

Follow-up monitoring in 2024 (the third year after establishment) revealed a continued and enhanced improvement trend (Table 3). SWC in the W3J1 treatment further increased to 27.28%, SEC declined further to 855.67 μS·cm^−1^, and pH was optimized to 7.44, indicating effective alleviation of soil salinization (*p* < 0.05). In terms of nutrient accumulation, SOM and TN reached 267.18 g·kg^−1^ and 7.35 g·kg^−1^, respectively, representing significant increases of 95.51 and 54.11% compared to CK (*p* < 0.05). However, TP content peaked in the W1J3 treatment (2.87 g·kg^−1^), with the W3J1 treatment ranking second (2.85 g·kg^−1^); both were significantly higher than CK (*p* < 0.05).

### 3.2 Effects of combined application of microbial inoculant and forage-specific fertilizer on soil bacterial community composition in artificial grassland

#### 3.2.1 Quality assessment of 16S sequencing data and OTU number variation

**Figure 1:**
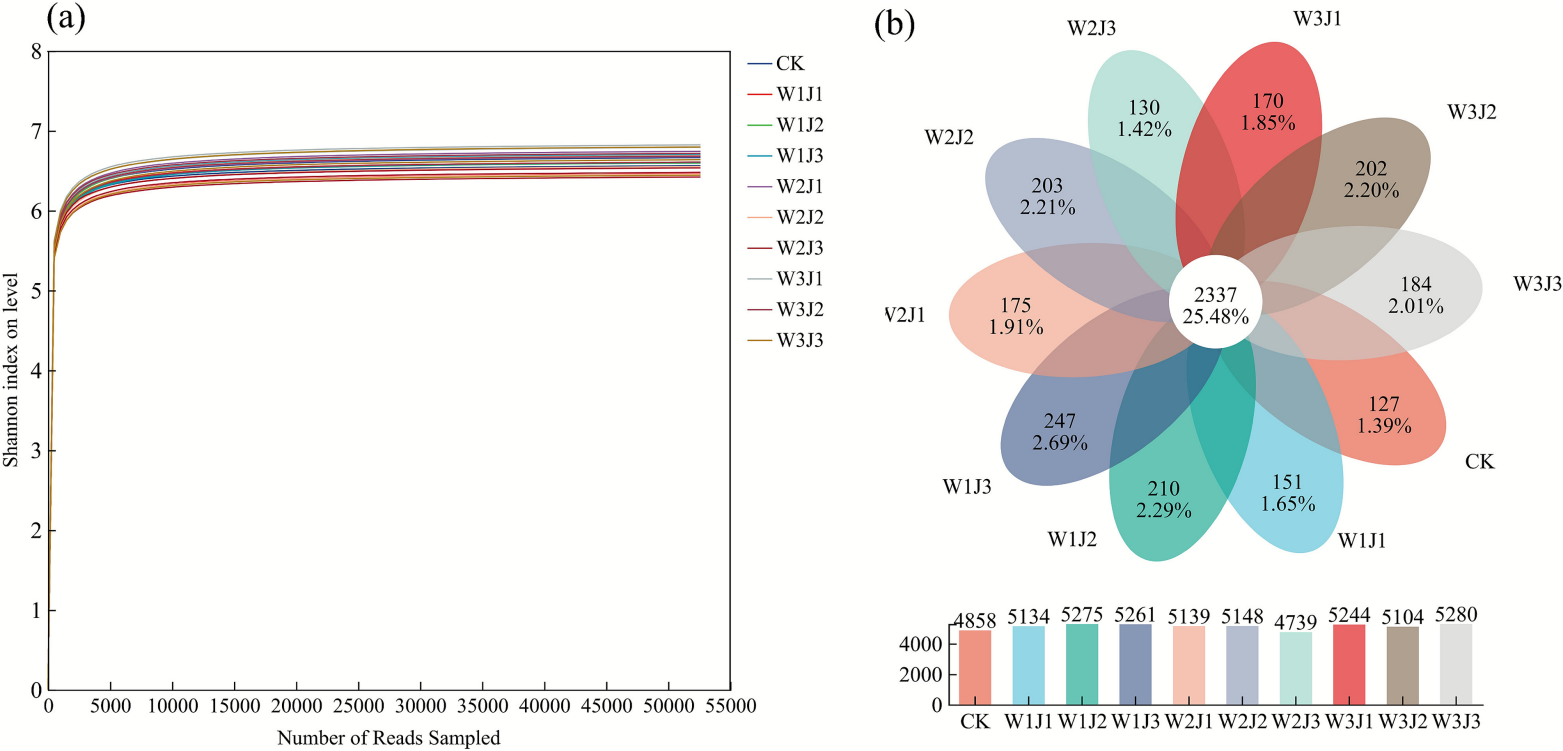
Rarefaction curves **(a)** and Venn diagram **(b)** of soil bacterial communities under different fertilization treatments. W denotes the application rate of forage-specific fertilizer (W1: 225.00 kg·hm^−2^, W2: 300.00 kg·hm^−2^, W3: 375.00 kg·hm^−2^), and J denotes the application rate of compound microbial inoculant (J1: 350.00 kg·hm^−2^, J2: 500.00 kg·hm^−2^, J3: 650.00 kg·hm^−2^).

The rarefaction curves showed that when the sequence sampling depth reached 5,000 reads, all treatment groups approached a clear plateau (Figure 1a), indicating that the current sequencing depth was sufficient to capture the majority of microbial diversity in the samples, and further increases in sequencing effort would yield limited gains in detecting new species. Venn diagram analysis revealed (Figure 1b) a total of 9,172 bacterial OTUs across the 10 treatment groups. The W3J3 and W1J2 treatments exhibited the highest species richness, with 5,280 and 5,275 OTUs, respectively, exceeding the control group (CK) value of 4,858 OTUs. In terms of unique OTUs, W1J3 displayed the highest number (247), followed by W1J2 (210), whereas CK had the lowest (127). Additionally, a total of 2,337 OTUs were shared among all treatment groups, accounting for only 25.48% of the total OTUs.

#### 3.2.2 Soil bacterial community composition and changes in relative abundance

**Figure 2:**
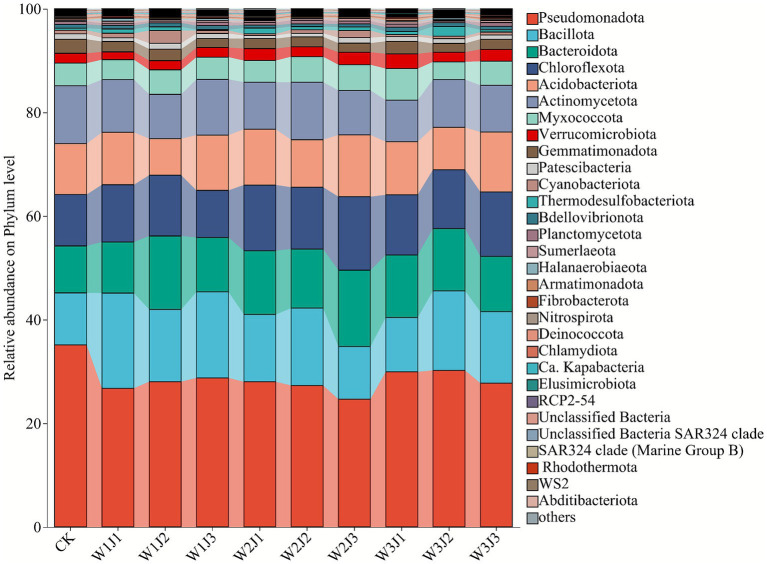
Relative abundance of soil bacterial communities at the phylum level under different fertilization treatments. W denotes the application rate of forage-specific fertilizer (W1: 225.00 kg·hm^−2^, W2: 300.00 kg·hm^−2^, W3: 375.00 kg·hm^−2^), and J denotes the application rate of compound microbial inoculant (J1: 350.00 kg·hm^−2^, J2: 500.00 kg·hm^−2^, J3: 650.00 kg·hm^−2^).

**Figure 3:**
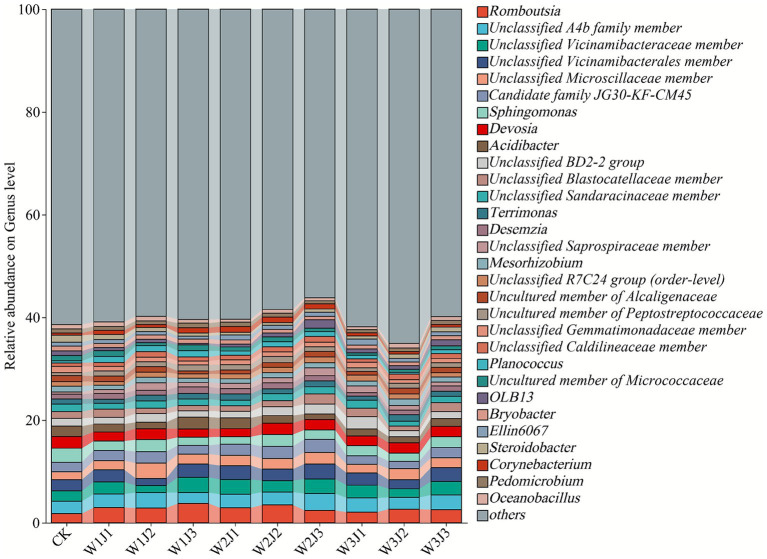
Relative abundance of soil bacterial communities at the genus level under different fertilization treatments. W denotes the application rate of forage-specific fertilizer (W1: 225.00 kg·hm^−2^, W2: 300.00 kg·hm^−2^, W3: 375.00 kg·hm^−2^), and J denotes the application rate of compound microbial inoculant (J1: 350.00 kg·hm^−2^, J2: 500.00 kg·hm^−2^, J3: 650.00 kg·hm^−2^).

As shown in Figure 2, relative abundance analysis at the phylum level revealed significant differences in the composition of dominant bacterial taxa across different fertilization treatments. The predominant bacterial phyla across all treatments were *Pseudomonadota* (24.67–35.15%), *Bacillota* (10.03–18.36%), *Bacteroidota* (9.06–14.72%), *Chloroflexi* (9.89–11.93%), *Acidobacteriota* (9.17–11.93%), and *Actinomycetota* (8.53–11.18%), all of which exhibited significantly higher relative abundances compared to other phyla. However, distinct differences in bacterial community structure were observed between the CK and fertilized treatments. Specifically, compared to CK, both the W2J3 and W3J1 treatments increased the relative abundance of *Bacteroidota* and *Chloroflexi*, while significantly decreasing the abundance of *Actinomycetota* (Figure 3).

**Figure 4:**
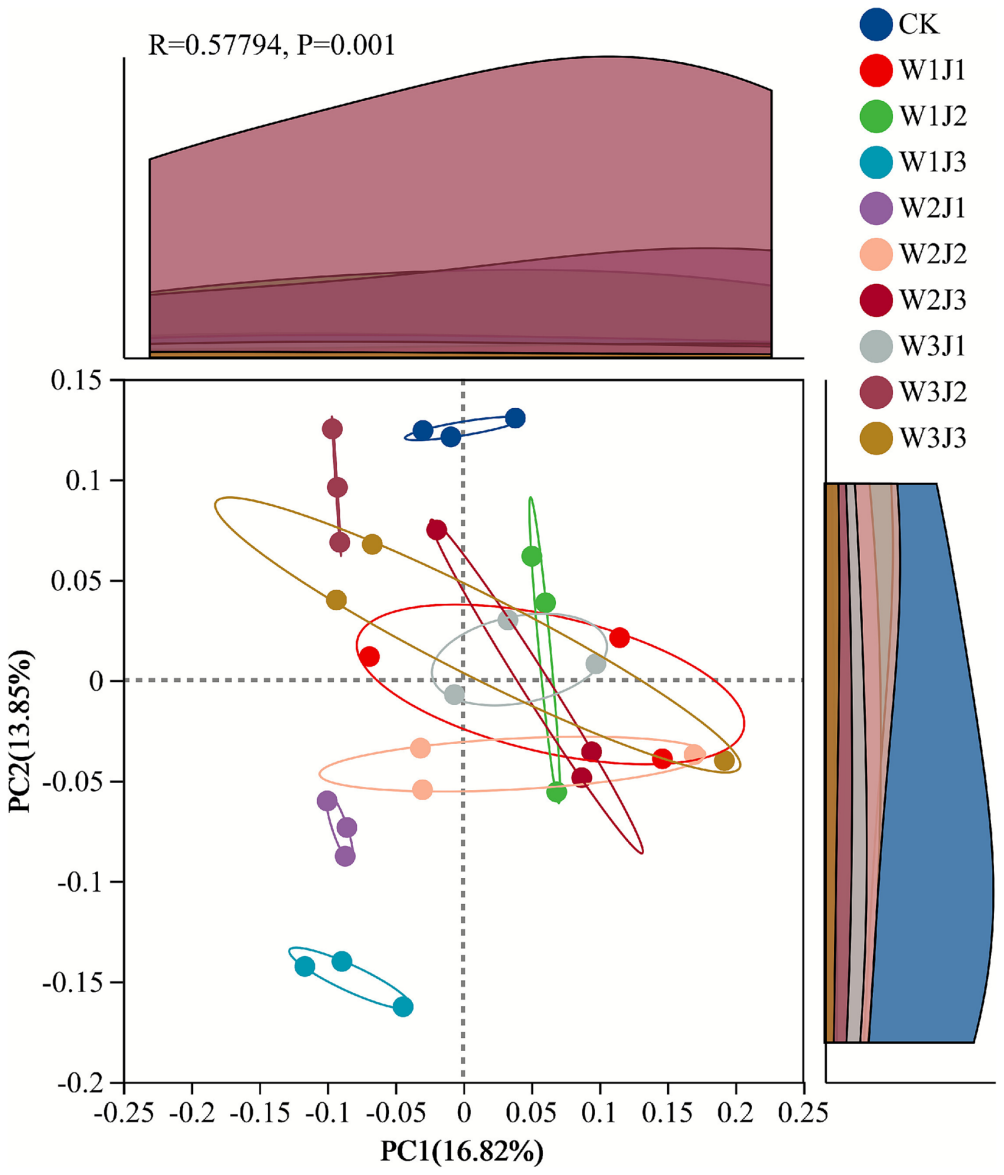
Differences in β-diversity of soil bacterial communities under different fertilization treatments, visualized by principal coordinates analysis (PCoA) based on Bray–Curtis dissimilarity. W denotes the application rate of forage-specific fertilizer (W1: 225.00 kg·hm^−2^, W2: 300.00 kg·hm^−2^, W3: 375.00 kg·hm^−2^), and J denotes the application rate of compound microbial inoculant (J1: 350.00 kg·hm^−2^, J2: 500.00 kg·hm^−2^, J3: 650.00 kg·hm^−2^).

At the genus level (Figure 4), the soil bacterial community was primarily composed of *Romboutsia* (1.78–3.75%), an unclassified member of the family A4b (1.38–2.99%), *Vicinamibacteraceae* (1.38–2.92%), *Vicinamibacterales* (1.55–2.98%), *Microscillaceae* (1.44–2.53%), *JG30-KF-CM45* (1.42–2.74%), and *Sphingomonas* (1.58–2.30%), all of which maintained relatively high abundances across treatments. Compared to the control, fertilizer treatments generally increased the relative abundance of *Romboutsia* (all >2.00%), with W1J3 and W2J2 showing the highest values (3.75 and 3.45%, respectively), significantly exceeding other treatments. In addition, fertilizer applications significantly reduced the abundance of the unclassified member of family JG30-KF-CM45.

#### 3.2.3 LEfSe analysis of soil bacterial communities

As shown in Supplementary Figure S3, linear discriminant analysis effect size (LEfSe) successfully identified microbial biomarkers that significantly contributed to differences in bacterial community structure across the 10 treatment groups (LDA score > 2.0). A total of 37 statistically significant discriminant taxa were detected across all treatments, with one feature identified in the W3J3 group and four in each of the other groups. At the taxonomic level, the dominant indicator taxa varied significantly among treatments: the CK treatment was characterized by *Gammaproteobacteria*; W1J1 by *Planococcaceae*; W1J2 by *Patescibacteria*; W1J3 by *Peptostreptococcales-Tissierellales*; W2J1 by *Corynebacteriaceae*; W2J2 by *Iamia*; W2J3 by *Anaerolineae*; W3J1 by *Burkholderiales*; W3J2 by *Anaerolineales*; and W3J3 by *Elsterales*.

### 3.3 Analysis of soil bacterial community diversity

#### 3.3.1 Soil bacterial community α-diversity

**Figure 5:**
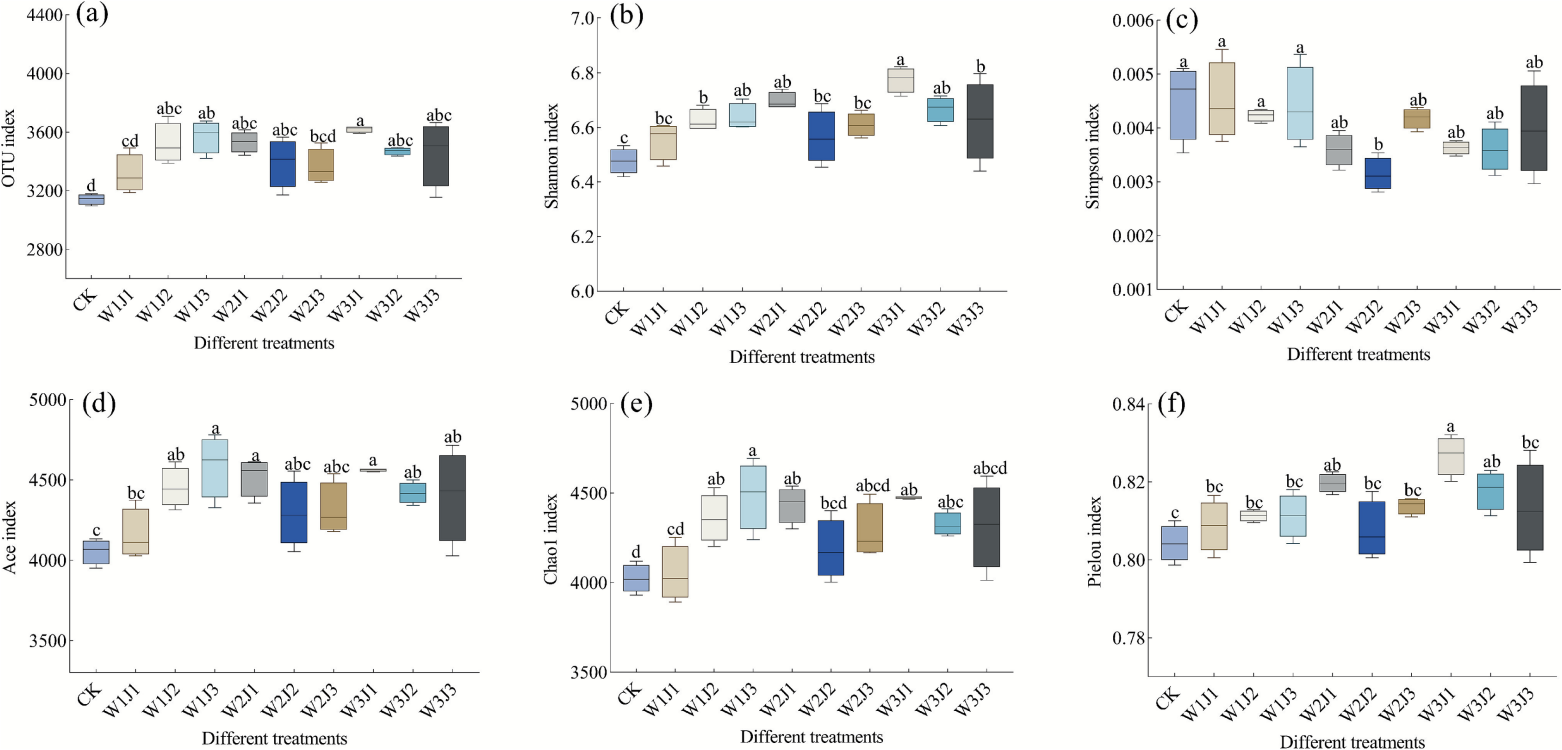
Differences in α-diversity indices of soil bacterial communities under different fertilization treatments. Panels show: OTU number **(a)**, Shannon index **(b)**, Simpson index **(c)**, Ace index **(d)**, Pielou index **(e)**, and Chao1 index **(f)**. W denotes the application rate of forage-specific fertilizer (W1: 225.00 kg·hm^−2^, W2: 300.00 kg·hm^−2^, W3: 375.00 kg·hm^−2^), and J denotes the application rate of compound microbial inoculant (J1: 350.00 kg·hm^−2^, J2: 500.00 kg·hm^−2^, J3: 650.00 kg·hm^−2^). Lowercase letters within panels indicate significant differences among treatments at *p* < 0.05.

As shown in Figure 5, α-diversity analysis indicated that the W3J1 treatment exhibited the best overall performance in terms of OTU number and community evenness. The OTU count (3,620), Shannon index (6.78), and Pielou’s evenness index (0.83) under this treatment were all significantly higher than those of the other treatments. In comparison, the corresponding values for the control group (CK) were 3,142, 6.48, and 0.80, respectively. Relative to CK, the W3J1 treatment increased the OTU count, Shannon index, and Pielou index by 15.21, 4.63, and 3.75%, respectively. In terms of species richness, the W1J3 treatment achieved the highest Ace (4,590) and Chao1 (4,487) index values, with the W3J1 treatment ranking second (4,562 and 4,475, respectively). Both were significantly higher than the CK treatment (*p* < 0.05), with respective increases of 13.16 and 12.48% for Ace, and 11.54 and 11.24% for Chao1. Additionally, the Simpson index across all treatments decreased in the following order: CK > W1J1 > W1J3 > W1J2 > W2J3 > W3J3 > W3J1 > W3J2 > W2J1 > W2J2. The W2J2 treatment had the lowest Simpson index, and the difference compared to the CK treatment was statistically significant (*p* < 0.05).

#### 3.3.2 β-diversity (PCoA) analysis of soil bacterial communities

Principal coordinates analysis (PCoA) based on Bray–Curtis dissimilarity revealed significant differences in soil bacterial community structure among the different treatment groups (*p* = 0.001; Figure 4). The first two principal coordinate axes (PC1 and PC2) explained 16.82 and 13.85% of the total variation, respectively, with a cumulative explained variance of 30.67%. Based on community structural similarity, the 10 treatment groups were clearly separated into three distinct clusters. The first cluster consisted solely of the CK treatment, with tightly clustered sample points indicating low within-group variation. The second cluster included only the W1J3 treatment. The third cluster comprised the remaining eight fertilized treatments. Among these, the W3J3, W1J1, and W2J2 treatments exhibited the greatest dispersion of sample points, indicating higher within-group variability, which suggested that these fertilizer combinations exerted a more pronounced influence on soil microbial community composition.

### 3.4 Changes in single-factor correlation networks of soil bacterial communities

**Figure 6:**
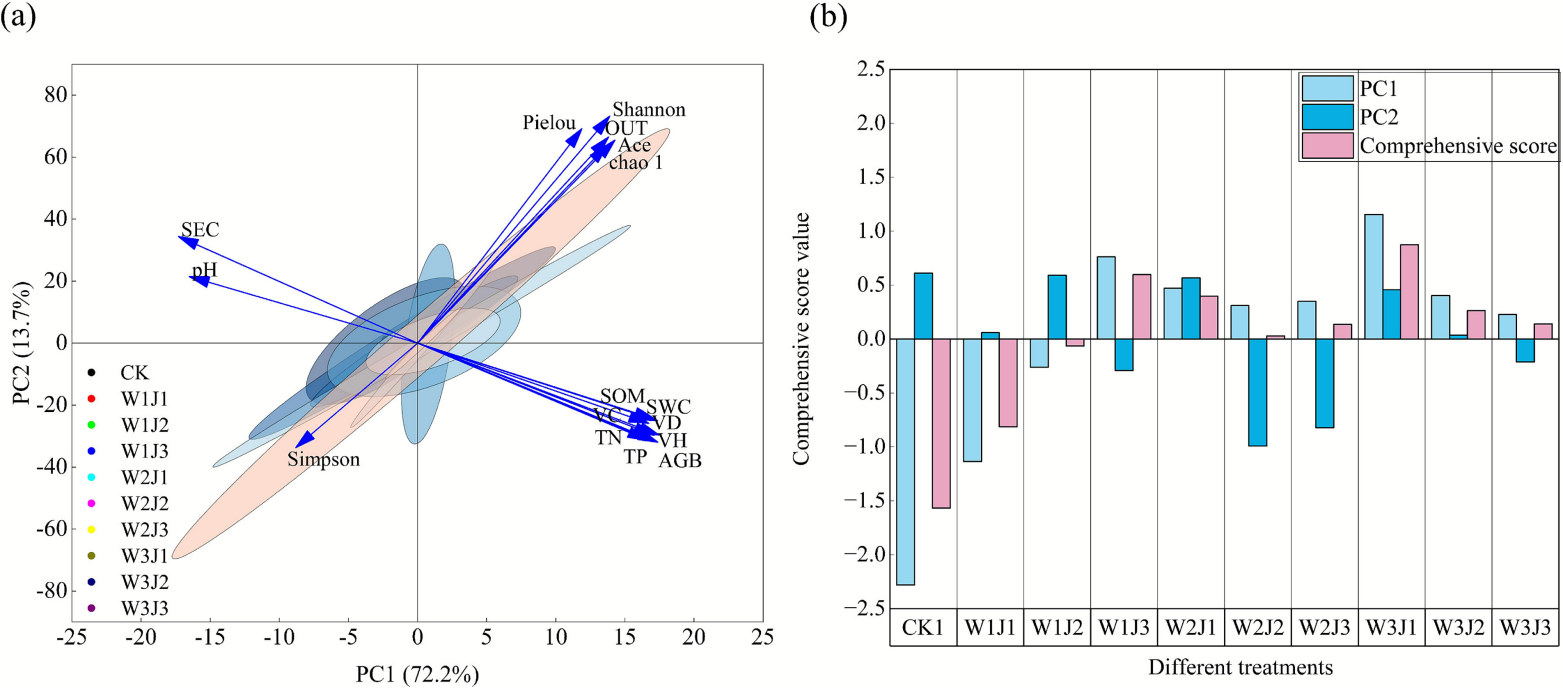
Integrated evaluation of soil physicochemical properties, plant community characteristics, and soil bacterial community structure using principal component analysis (PCA). Panel **(a)** presents the PCA biplot of variables and treatment groups; panel **(b)** shows the comprehensive PCA score plot across treatments. W denotes the application rate of grass-specific fertilizer (W1: 225.00 kg·hm^−2^, W2: 300.00 kg·hm^−2^, W3: 375.00 kg·hm^−2^), and J denotes the application rate of composite microbial inoculant (J1: 350.00 kg·hm^−2^, J2: 500.00 kg·hm^−2^, J3: 650.00 kg·hm^−2^). VH, vegetation height; VC, vegetation cover; VD, vegetation density; AGB, aboveground biomass; SWC, soil water content; SEC, soil electrical conductivity; pH, soil pH; SOM, soil organic matter; TN, total nitrogen; TP, total phosphorus.

To gain deeper insights into the effects of different fertilization treatments on microbial inter-taxa interactions, bacterial co-occurrence networks were constructed at the genus level (Supplementary Figures S4, S5). The results indicated that fertilization treatments significantly altered the topological structure of the soil bacterial networks. The number of network connections (edges) varied markedly among treatments, ranging from 700 to 1,252. The W1J2 treatment exhibited the highest number of edges (1,252), which was significantly greater than that of the W1J1 treatment (816). In contrast, the number of network nodes remained relatively stable across all treatments, ranging between 49 and 50. Furthermore, the proportion of positive correlations in the bacterial networks differed significantly among treatments. The W2J1 treatment showed the highest proportion of positive correlations (57.58%), which was notably higher than that of the CK treatment (48.80%). Single-factor correlation network analysis (Figure 6) revealed that, across the nine fertilized treatments, six phyla, *Pseudomonadota*, *Chloroflexota*, *Bacillota*, *Bacteroidota*, *Actinomycetota*, and *Myxococcota*, served as core hub taxa with high connectivity. In contrast, in the CK group, *Cyanobacteriota* and *Verrucomicrobiota* played key roles in network connectivity.

### 3.5 Functional prediction of soil bacterial communities

**Figure 7:**
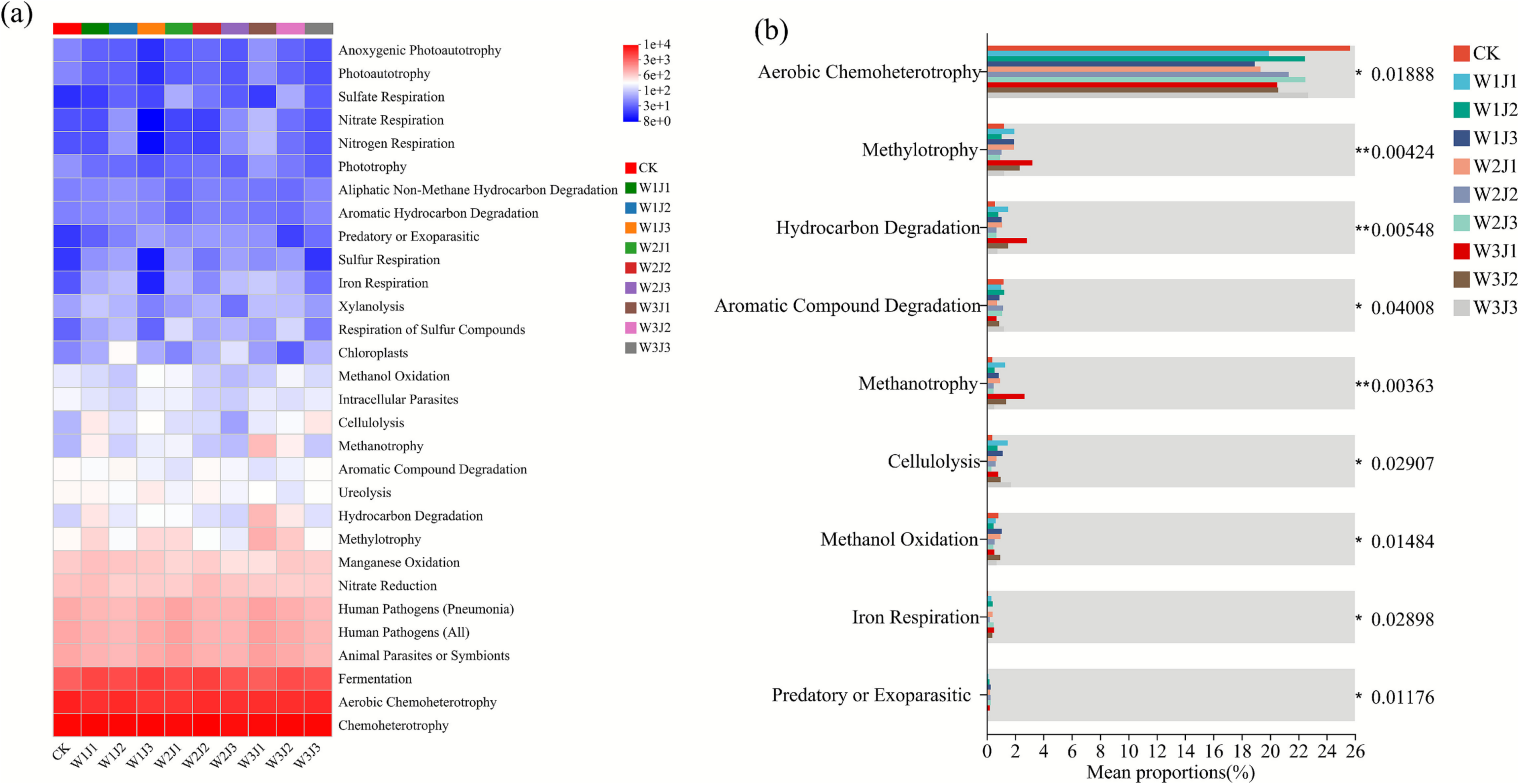
Functional prediction of soil bacterial communities using FAPROTAX and intergroup significance analysis across fertilization treatments. Panel **(a)** shows the heatmap of predicted functional profiles; panel **(b)** presents the significant differences among treatment groups based on FAPROTAX-predicted functional annotations. W denotes the application rate of forage-specific fertilizer (W1: 225.00 kg·hm^−2^, W2: 300.00 kg·hm^−2^, W3: 375.00 kg·hm^−2^), and J denotes the application rate of compound microbial inoculant (J1: 350.00 kg·hm^−2^, J2: 500.00 kg·hm^−2^, J3: 650.00 kg·hm^−2^).

Based on FAPROTAX functional prediction analysis, this study systematically characterized the functional profiles of soil bacterial communities under 10 fertilization treatments. As shown in Figure 7a, a total of 30 metabolic functions were identified across all treatments, with chemoheterotrophy, aerobic chemoheterotrophy, and fermentation being the dominant metabolic pathways. Specifically, the W1J3 and W2J2 treatments exhibited the highest relative abundances of chemoheterotrophy (10,132 and 10,471, respectively) and fermentation (4,499 and 4,327, respectively), both of which were substantially higher than in the CK treatment (2,779), representing increases of 54.7 to 61.8%. Additionally, the W3J1 treatment displayed a distinct metabolic profile, showing higher abundances of hydrocarbon degradation (813), methylotrophy (915), and methanotrophy (770) compared to all other treatments.

Functional difference analysis further revealed that the W1J3 treatment had the lowest functional abundances of anoxygenic photoautotrophy (15.67) and photoautotrophy (15.67) among all treatments. The potential for nitrate respiration (8.33) and nitrogen respiration (9.33) in this treatment was also lower than in other treatments. Except for W3J1, the CK treatment exhibited the lowest abundance of sulfate respiration (15.67), significantly lower than all fertilized treatments.

As shown in Figure 7b, the functional group comparison analysis demonstrated that aerobic chemoheterotrophy was the predominant metabolic function in the microbial communities, with significantly higher relative abundances in the CK, W1J2, W2J3, and W3J3 treatments compared to other functional groups. Although methylotrophy was generally low in relative abundance (<6%), it was notably enriched in the W3J1 and W3J2 treatments, suggesting that these treatments may promote the growth of methylotrophic bacterial populations. Furthermore, hydrocarbon degradation and methanotrophy functions exhibited low abundances across most treatments (<4%), but showed increased levels in W3J1 and W3J2. These results indicated that different fertilization treatments significantly affected the functional structure of soil microbial communities by modulating resource availability.

### 3.6 Hierarchical clustering analysis of soil bacterial communities

Hierarchical clustering analysis based on the Bray–Curtis dissimilarity matrix revealed a significant effect of different fertilization treatments on soil bacterial community structure (Supplementary Figure S6). At a similarity threshold of 0.19, all samples were clearly separated into two distinct clusters: Cluster 1 consisted of the W1J1, W1J2, W2J2, and W3J3 treatments, while Cluster 2 included the CK control and the remaining treatment groups. Further analysis showed that when the similarity threshold was reduced to 0.17, Cluster 2 could be subdivided into two significantly differentiated subclusters: one subcluster comprised the CK treatment, W1J1, W2J3, W3J2, and W3J3, whereas the other subcluster grouped the remaining fertilized treatments. Additionally, the CK treatment exhibited high within-group similarity and was clearly distinct from all other treatment groups.

### 3.7 Coupling relationships among vegetation community characteristics, soil physicochemical properties, and soil bacterial communities

#### 3.7.1 Mantel test analysis of vegetation, soil properties, and soil bacterial communities

**Figure 8:**
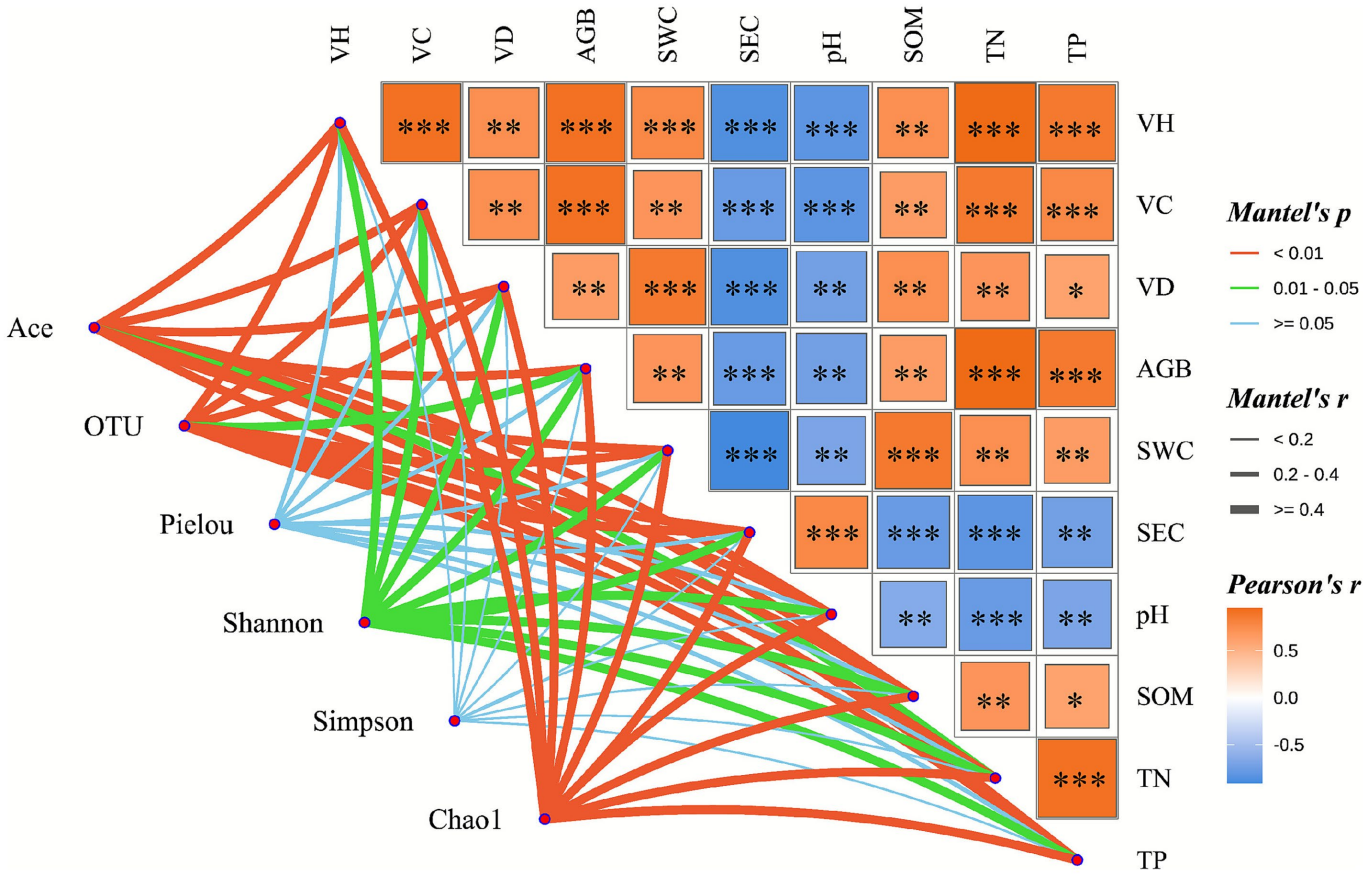
Mantel test analysis of the relationships among vegetation community characteristics, soil physicochemical properties, and soil bacterial communities. VH, vegetation height; VC, vegetation cover; VD, vegetation density; AGB, aboveground biomass; SWC, soil water content; SEC, soil electrical conductivity; pH, soil pH; SOM, soil organic matter; TN, total nitrogen; TP, total phosphorus.

Mantel test results assessing environmental factor associations (Figure 8) revealed an extremely significant positive correlation between soil pH and SEC (*p* < 0.001). However, both pH and SEC showed highly significant negative correlations with all other soil physicochemical properties and vegetation characteristics (*p* < 0.01). With respect to vegetation attributes, vegetation density was significantly positively correlated only with TP content (*p* < 0.05), but exhibited highly significant positive correlations with all other vegetation indices and soil parameters (*p* < 0.01). Further analysis indicated that vegetation height, vegetation cover, and aboveground biomass were extremely significantly positively correlated with both TN and TP contents (*p* < 0.001) and highly significantly correlated with SOM content (*p* < 0.01).

The bacterial community Ace index was significantly correlated only with TN content (*p* < 0.05), while showing highly significant correlations with all other soil physicochemical properties and vegetation characteristics (*p* < 0.01). In terms of species richness, OTU number was significantly correlated with aboveground biomass (*p* < 0.05) and highly significantly correlated with all other environmental variables (*p* < 0.01). In contrast, community evenness indices (Pielou evenness and Simpson index) showed no significant correlations with any of the measured variables (*p* > 0.05), whereas the Shannon diversity index was significantly associated with all tested indicators (*p* < 0.05). The Chao1 richness index exhibited highly significant correlations with both vegetation characteristics and soil physicochemical parameters (*p* < 0.01).

#### 3.7.2 Redundancy analysis (RDA) of vegetation community characteristics, soil physicochemical properties, and soil bacterial communities

**Figure 9:**
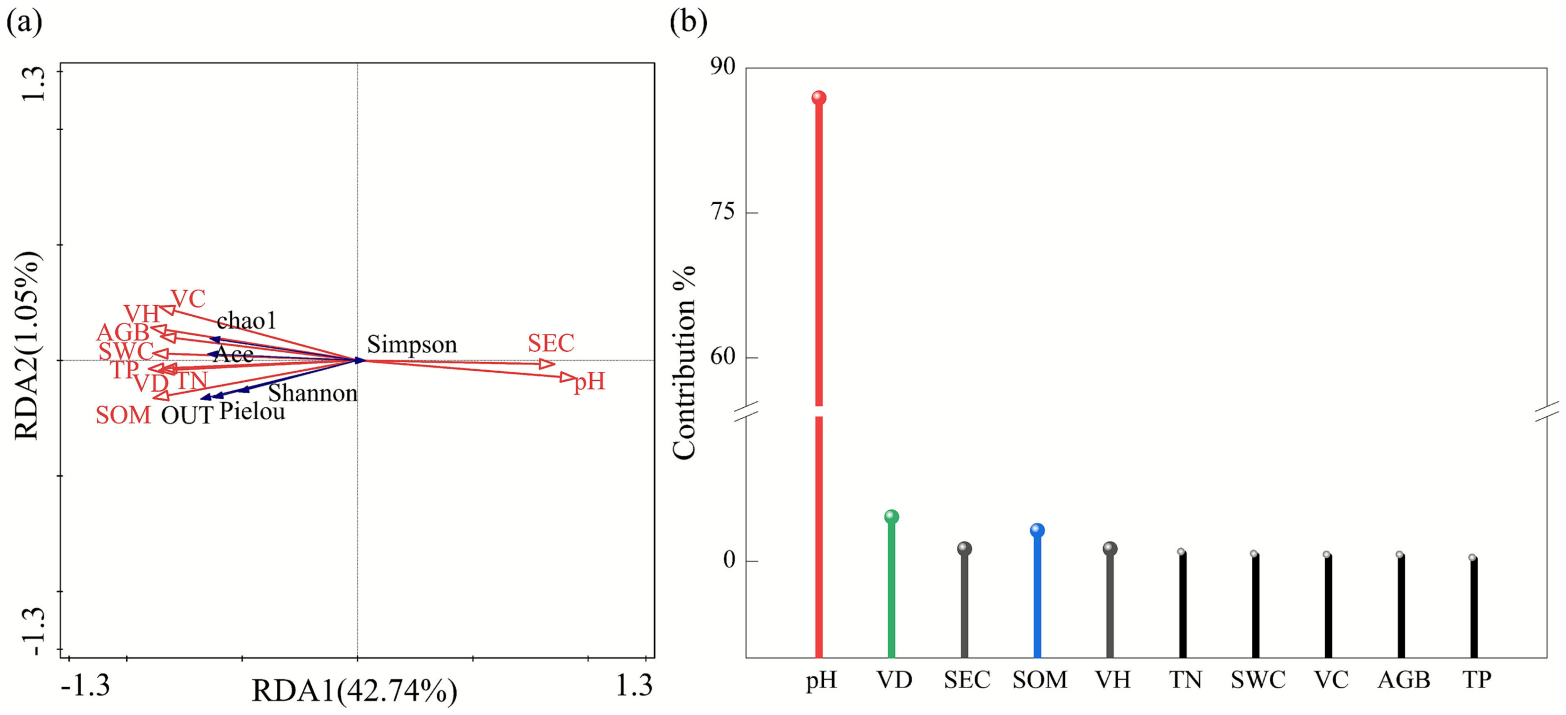
Redundancy analysis (RDA) of the relationships among soil physicochemical properties, plant community characteristics, and soil bacterial community structure. Panel **(a)** shows the RDA ordination plot of bacterial community composition in relation to environmental variables; panel **(b)** presents the contribution rates of individual environmental factors. VH, vegetation height; VC, vegetation cover; VD, vegetation density; AGB, aboveground biomass; SWC, soil water content; SEC, soil electrical conductivity; pH, soil pH; SOM, soil organic matter; TN, total nitrogen; TP, total phosphorus.

Redundancy analysis (RDA) revealed significant relationships between soil bacterial community diversity and both vegetation characteristics and soil physicochemical factors (Figure 9). The first two ordination axes (Axis I and Axis II) explained 42.74 and 1.05% of the total variation, respectively, with a cumulative explained variance of 43.79%. Environmental variable partitioning showed that soil pH had the most significant influence on bacterial community diversity, accounting for 38.20% of the explained variation and contributing 86.90% to the overall environmental factor effects. Soil pH was positively correlated with the Simpson index but showed significant negative correlations with the Shannon index, OTU number, Ace index, and Chao1 index. In contrast, all other soil variables, including SOM, TN, and TP contents, and vegetation parameters were significantly positively correlated with bacterial diversity indices, except for the Simpson index.

#### 3.7.3 Path analysis of vegetation community characteristics, soil physicochemical properties, and soil bacterial communities

**Figure 10:**
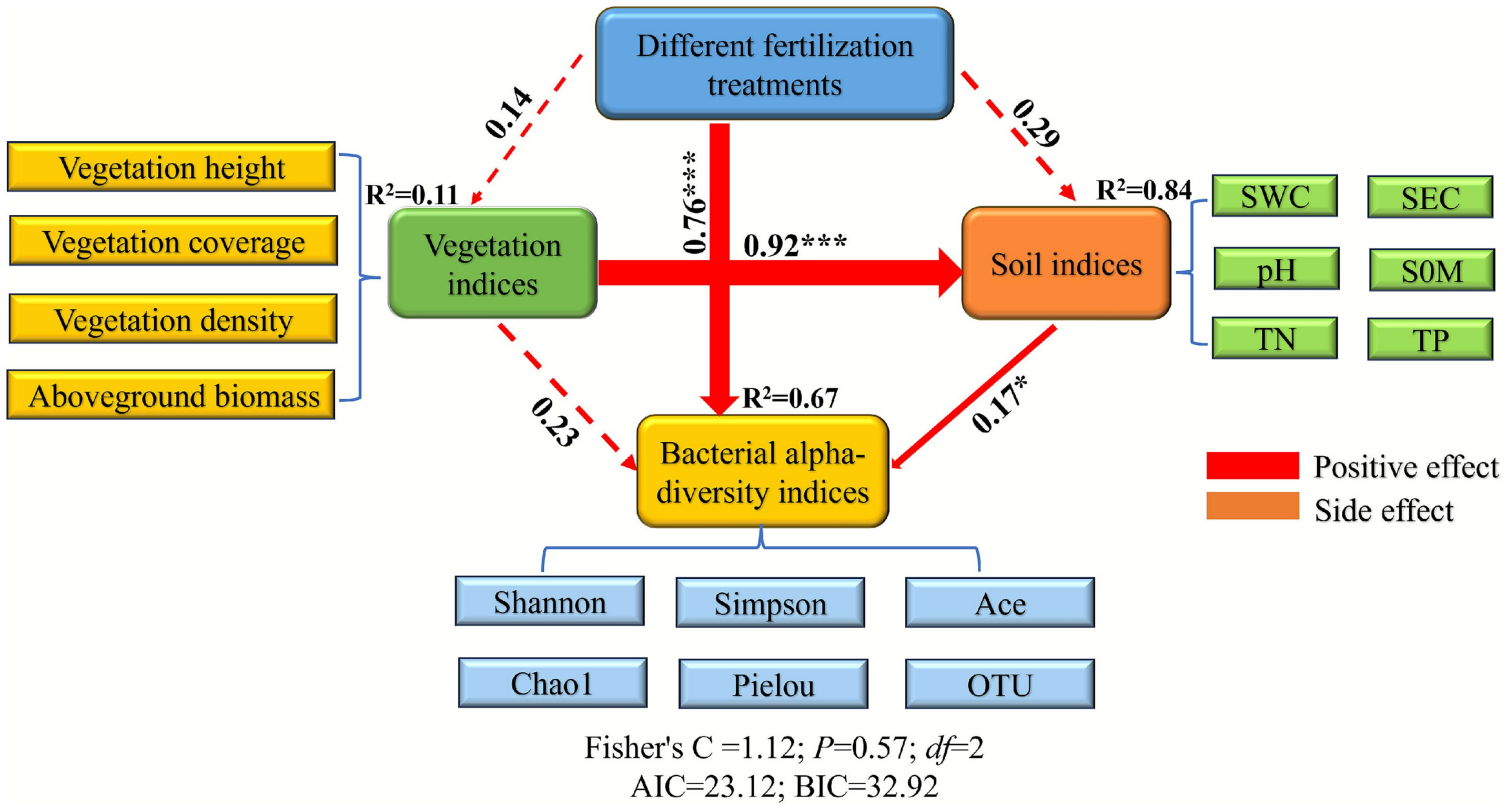
Path analysis of the relationships among fertilization treatments, vegetation community characteristics, soil physicochemical properties, and soil bacterial α-diversity. Solid lines indicate significant path coefficients, dashed lines indicate non-significant paths; red lines represent positive, and orange lines represent negative, correlations. Numbers adjacent to lines denote standardized path coefficients (β), with line width proportional to the magnitude of the coefficient. SWC, soil water content; SEC, soil electrical conductivity; pH, soil pH; SOM, soil organic matter; TN, total nitrogen; TP, total phosphorus.

Structural equation modeling (SEM) was employed to systematically elucidate the regulatory pathways of fertilization treatments on soil bacterial communities in alpine grassland ecosystems (Figure 10). The model exhibited good fit to the data (Fisher’s *C* = 1.12, *p* = 0.57, *df* = 2, AIC = 23.12, BIC = 32.92). The results revealed that fertilization treatments exerted a strong direct positive effect on bacterial *α*-diversity (*β* = 0.76, *p* < 0.001), but showed no significant direct effects on vegetation indices (*β* = 0.14, *p* > 0.05) or soil physicochemical properties (*β* = 0.29, *p* > 0.05). Further analysis indicated that vegetation indices had a strong positive driving effect on soil physicochemical properties (*β* = 0.92, *p* < 0.001), yet their direct influence on bacterial diversity was not significant (*β* = 0.23, *p* > 0.05). In contrast, soil physicochemical properties significantly promoted bacterial diversity (*β* = 0.17, *p* < 0.05). Variance partitioning showed that the path model explained 67% of the variation in bacterial α-diversity (*R*^2^ = 0.67). Therefore, fertilization primarily acted directly on microbial communities, while also indirectly modulating bacterial diversity through a “vegetation → soil” cascading pathway.

#### 3.7.4 Integrated evaluation analysis of vegetation community characteristics, soil physicochemical properties, and soil bacterial communities

Principal component analysis (PCA) was applied to systematically reduce the dimensionality of 16 indicators encompassing vegetation community characteristics, soil physicochemical properties, and bacterial community features (Figure 6a). The Kaiser–Meyer–Olkin (KMO) measure of sampling adequacy was 0.82 (*p* < 0.001), indicating that the dataset was highly suitable for PCA. The analysis revealed that the first two principal components (PC1 and PC2) collectively explained 85.90% of the total variance. Specifically, PC1, with an eigenvalue of 11.55, accounted for 72.20% of the variation in the original dataset, while PC2 explained an additional 13.70%. This high cumulative explained variance indicated substantial systemic correlations among the ecological variables.

Principal component scores (Figure 6b) revealed significant differences in ecological restoration performance across fertilization treatments. The W3J1 treatment exhibited the highest score along PC1 (1.16), followed by W1J3 (0.76), indicating superior overall ecological improvement. Along PC2, the CK control (0.61) and W1J2 treatment (0.59) showed relatively high scores, which reflected distinct ecological response patterns. Based on the weighted comprehensive scores derived from principal components, the overall ranking of ecosystem restoration efficacy among the treatments was: W3J1 > W1J3 > W2J1 > W3J2 > W3J3 > W2J3 > W2J2 > W1J2 > W1J1 > CK.

## 4 Discussion

### 4.1 Mechanisms of soil amelioration by the combined application of composite microbial inoculant and forage-specific fertilizer in artificial grasslands of alpine mining areas

This study systematically elucidated the synergistic amelioration mechanisms of the combined application of composite microbial inoculant and forage-specific fertilizer in artificial grasslands established in alpine mining regions. The microbial inoculant introduces exogenous microbial communities with functional traits such as phosphorus solubilization and nitrogen fixation, which complement the nitrogen, phosphorus, and potassium supplied by the forage-specific fertilizer. This interaction enhances the conversion of soil nutrients into bioavailable forms (Deng et al., 2025). Results showed that the optimized treatment (W3J1) significantly increased grassland productivity, with aboveground biomass rising by 72.78 and 80.23% compared to the CK control in 2023 and 2024, respectively. These findings are consistent with the plant growth-promoting mechanisms proposed by Prabawardani et al. (2023). However, a clear threshold effect in application rates was also observed: excessive inputs (as in the W3J3 treatment) led to microbial community imbalance and intensified nutrient competition, ultimately inhibiting vegetation growth—results that align with those reported by Song et al. (2015) and Cai et al. (2018).

From the perspective of soil physical properties, the W3J1 treatment maintained SWC above 26% for two consecutive years, outperforming the soil amendment effects of manure reported by Zhang H. J. et al. (2025) and Zhang Y. F. et al. (2025). This enhanced water retention capacity was likely attributable to improved soil aggregate structure mediated by microbial metabolites such as extracellular polysaccharides, thereby increasing soil water-holding capacity (Qurashi and Sabri, 2012). Regarding chemical property improvement, the W3J1 treatment reduced soil electrical conductivity to 875.67–855.67 μS·cm^−1^ and stabilized pH within the favorable range of 7.77–7.44, consistent with findings by Puy Alquiza et al. (2025) on microbial inoculant-mediated soil amelioration in mining areas. These results suggested that organic acids secreted by microorganisms neutralize alkaline substances, effectively mitigating the risk of secondary salinization commonly associated with conventional chemical fertilizer application. In terms of nutrient accumulation, the W3J1 treatment increased SOM and TN contents by 95.51 and 54.11%, respectively, which aligned with Su et al. (2025), who reported that microbial inoculants promote organic matter accumulation, likely due to enhanced microbial biomass and accelerated cycles of organic matter decomposition and synthesis. Although the W1J3 treatment exhibited slightly higher TP accumulation than W3J1, the latter demonstrated superior overall performance in salinity-alkalinity regulation and coordinated enhancement of carbon and nitrogen.

Notably, the W3J3 treatment, characterized by excessive application, led to a rebound in soil salinization, as indicated by increased electrical conductivity and pH, which revealed that over-fertilization disrupts the soil’s buffering capacity (Singh et al., 2025), a finding consistent with that of Chu (2014). This phenomenon fundamentally arose when excessive nutrient inputs exceeded the metabolic capacity of the microbial community, leading to salt accumulation. These results not only confirmed the synergistic effects of combined microbial inoculant and forage-specific fertilizer application but also defined their operational boundaries, providing critical theoretical insights and technical parameters for the precise restoration of degraded grasslands in alpine mining ecosystems.

### 4.2 Effects of combined application of composite microbial inoculant and forage-specific fertilizer on soil bacterial community structure and function in artificial grasslands

This study reveals the regulatory effects of the combined application of composite microbial inoculant and forage-specific fertilizer on soil bacterial community structure in alpine mining areas, with findings that both corroborate previous studies and highlight regional specificity (Zhang et al., 2020; Xu et al., 2025). At the phylum level, the distribution patterns of dominant bacterial groups, including *Pseudomonadota*, *Bacillota*, and *Bacteroidota*, are consistent with those reported by Zhang et al. (2020) in microbial communities of coal mine reclamation areas, confirming the ecological adaptability of these taxa to degraded mining soils. Furthermore, treatments W2J3 and W3J1 increased the relative abundances of *Bacteroidota* and *Chloroflexi*, a shift primarily attributed to the colonization of functional microbial taxa introduced via the composite inoculant and the essential nitrogen, phosphorus, and potassium supplied by the forage-specific fertilizer. This observation provides mutual support for the nutrient-selective regulation theory proposed by Xue et al. (2025). Concurrently, the reduced abundance of *Actinobacteriota* reflects a shift in soil microenvironmental conditions from oligotrophic to *copiotrophic* states induced by fertilization. This response pattern contrasts with findings from Xu et al. (2025) in agricultural ecosystems, underscoring the influence of ecosystem-specific factors on microbial community assembly.

At the genus level, the study confirmed that seven genera, including *Romboutsia*, constitute the core components of the bacterial community. While this finding shared commonality with Hou (2022) regarding microbial responses to inoculant-fertilizer combinations in degraded soils of northwestern Liaoning, the composition of dominant taxa exhibited distinct regional characteristics. Treatments W1J3 and W2J2 significantly increased the relative abundance of *Romboutsia* to 3.75 and 3.45%, respectively, which may be linked to mutualistic interactions between functional strains in the microbial inoculant and *Romboutsia*, as well as improved soil physicochemical conditions under optimized fertilization regimes that favor its proliferation (Kampouris et al., 2025). Additionally, fertilization treatments significantly suppressed the abundance of an uncultured and unclassified bacterial lineage within the family-level group *JG30-KF-CM45*. This decline may result from the group’s sensitivity to environmental changes, competitive disadvantage for resources, and potential antagonistic interactions with introduced functional taxa. This finding contrasts with observations by Gao et al. (2025) in wetland ecosystems, further confirming the unique response mechanisms of microbial communities in alpine mining environments.

Bacteria, as key components of soil microbial communities, profoundly influence crop growth, soil quality, and ecosystem health through changes in their rhizospheric structure and diversity (Yao et al., 2025). The application of microbial inoculants has been demonstrated to exert positive effects on soil microbial community structure and diversity (Liu T. N. et al., 2025; Liu M. et al., 2025; Liu H. H. et al., 2025; Liu Q. Q. et al., 2025). Wang et al. (2024) reported that microbial inoculant application altered soil microbial diversity in tomato cultivation systems and significantly increased the bacterial Chao1 and Shannon indices. Ma et al. (2020) observed enhanced bacterial diversity and evenness in greenhouse eggplant soils following microbial inoculant application. In this study, the W3J1 treatment exhibited the most pronounced improvement in microbial diversity, with OTU number, Shannon index, and Pielou index increased by 15.21, 4.63, and 3.75%, respectively, relative to the CK control. This finding is consistent with Ollio et al. (2025), who reported enhanced soil microbial diversity following microbial inoculant application. Moreover, W3J1 maintained high species richness (with Ace and Chao1 indices slightly lower than those under W1J3) while significantly improving community evenness (highest Pielou index), indicating that this fertilization regime promotes both species coexistence and functional optimization of the soil microbial community (Liu T. N. et al., 2025; Liu M. et al., 2025; Liu H. H. et al., 2025; Liu Q. Q. et al., 2025). In terms of species richness, the W1J3 and W3J1 treatments increased the indices by 13.16 and 11.54%, respectively, compared to CK, a result corroborated by Yang et al. (2021), further confirming that optimized microbial inoculant–fertilizer combinations can establish more stable microbial community structures. This study also revealed that excessively high rates of microbial inoculant and forage-specific fertilizer application (e.g., W3J3 treatment) led to reduced microbial diversity, a phenomenon consistent with the “intermediate ratio effect” theory, suggesting a threshold response in microbial community dynamics (Wei et al., 2023). Simpson index analysis further showed that the W2J2 treatment had the lowest dominance, with a significant difference compared to CK (*p* < 0.05), likely due to the synergistic optimization of microenvironmental physicochemical properties by microbial inoculant and forage-specific fertilizer, as well as balanced nutrient inputs that mitigate interspecific competition (Ma et al., 2020).

Co-occurrence network analysis revealed the impact of fertilization treatments on microbial intertaxa interactions in alpine mining soils. Compared with the findings of Duan et al. (2025), the bacterial co-occurrence network in this alpine mining ecosystem exhibited higher topological complexity, with 700 to 1,252 edges detected across treatments, which may reflect an enhanced requirement for functional complementarity among microorganisms under extreme environmental conditions (Liu T. N. et al., 2025; Liu M. et al., 2025; Liu H. H. et al., 2025; Liu Q. Q. et al., 2025). Regarding the proportion of positive correlations, the W2J1 treatment showed a significantly higher percentage of positive links (57.58%) than the CK control (48.80%). This shift may be attributed to two underlying mechanisms: (1) fertilization alleviated resource limitations, thereby reducing competitive pressures among microbial taxa; and (2) the introduction of specific functional microbial groups altered interaction patterns among species, a finding consistent with Wu et al. (2025) on microbial cooperative metabolism. In keystone taxon identification, six phyla, including *Pseudomonadota* and *Chloroflexota*, were identified as central hubs in the fertilized soils, a result that contrasts with the findings of Shi et al. (2025). In contrast, the critical roles of *Cyanobacteria* and *Verrucomicrobiota* in the CK control reflect the unique niche allocation patterns characteristic of the original, unamended soil. The shift in dominant hub taxa induced by fertilization indicates that anthropogenic intervention substantially altered the assembly mechanisms of the soil microbial community (Liu T. N. et al., 2025; Liu M. et al., 2025; Liu H. H. et al., 2025; Liu Q. Q. et al., 2025).

FAPROTAX functional prediction results revealed that chemoheterotrophy, aerobic chemoheterotrophy, and fermentation constituted the core components of the soil microbial metabolic network, a finding consistent with Li et al. (2025) in their study on the regulation of soil microbial functions by combined application of composite microbial inoculant and biochar in degraded grasslands. Under different fertilizer ratios, the W1J3 and W2J2 treatments exhibited the highest abundances of chemoheterotrophy (10,471) and fermentation (4,499), representing increases of 54.7–61.8% compared to the CK control. This enhancement may be attributed to the activation of heterotrophic microbial metabolic activity by nitrogen, phosphorus, and potassium supplied through the forage-specific fertilizer, coupled with optimized microbial growth conditions under specific inoculant formulations, closely aligning with the “nitrogen source–microbe” synergistic effect theory proposed by Hu et al. (2021). The W3J1 treatment displayed a distinct metabolic profile, with significant enrichment in hydrocarbon degradation (813), methylotrophy (915), and methanotrophy (770), reflecting selective enrichment of microbial taxa with specialized metabolic capabilities by specific components of the forage-specific fertilizer, as well as functional community restructuring mediated by the introduced microbial inoculant, phenomena similar to those reported by Xiao et al. (2021) in functional regulation of mine-affected soils. Furthermore, the W1J3 treatment showed significantly reduced abundances of photoautotrophy and nitrogen respiration, a result contrasting with the findings of Huang et al. (2019) regarding the impact of inorganic fertilization on bacterial functions in paddy soils, highlighting the unique metabolic adaptation of alpine mining soil microorganisms, such as optimization of energy allocation, in response to fertilizer regimes (Zhang H. J. et al., 2025; Zhang Y. F. et al., 2025).

It should be noted that microbial functions were predicted from 16S rRNA gene data using FAPROTAX, which infers potential metabolic capabilities based on taxonomy. While this approach offers insights into functional trends, it has limitations: predictions rely on incomplete reference databases, may misrepresent functions in understudied environments, and do not reflect actual gene expression or activity. Moreover, strain-level variation and horizontal gene transfer are not captured, and inferred functions may not equate to *in situ* activity. Thus, the results represent *potential* rather than *active* microbial functions (Sansupa et al., 2021).

For accurate functional profiling, future studies should employ shotgun metagenomics to directly identify functional genes and integrate metatranscriptomics and metabolomics to validate gene expression and metabolic activities (Djemiel et al., 2022). Although functional predictions were inferred rather than directly measured, the observed shifts align with improvements in soil properties and plant growth, suggesting potential ecological relevance. However, caution is warranted when interpreting taxonomy-based functional predictions, particularly in extreme ecosystems such as alpine mining areas.

### 4.3 Interactions among rhizosphere microbial communities, environmental factors, and vegetation characteristics, and integrated assessment of fertilizer regulation effects

Mantel tests and redundancy analysis (RDA) were employed to systematically elucidate the interaction mechanisms within the rhizosphere microbe–soil–vegetation system in the alpine mining area. Mantel test results revealed an extremely significant positive correlation between soil pH and electrical conductivity. Vegetation community characteristics (including height, coverage, and density), soil nutrient indicators (soil organic matter, total nitrogen, and total phosphorus), and soil water content all showed significant positive correlations with microbial community composition. These findings are consistent with those of Qin et al. (2025) on the effects of nitrogen and phosphorus addition in degraded grasslands of the Qinghai–Tibet Plateau, further confirming that soil nutrient availability (particularly nitrogen and phosphorus) and water availability are key limiting factors governing plant community development in alpine ecosystems (Zhang H. J. et al., 2025; Zhang Y. F. et al., 2025). Responses of bacterial diversity indices to environmental factors exhibited distinct differences: the Ace index was significantly correlated only with total nitrogen (TN), whereas the Chao1 index showed significant correlations with all tested variables. This discrepancy may reflect varying sensitivities of diversity indices to changes in microbial community structure, a phenomenon previously reported by Li et al. (2021) in soil restoration studies of open-pit coal mining areas. RDA further demonstrated that soil pH was the dominant driver of bacterial community diversity variation, explaining 38.20% of the variance. This result aligns with findings from Cao et al. (2020) in coal mining areas of Shanxi, China, and may be attributed to the direct influence of pH on microbial metabolism through modulation of cell membrane permeability and enzyme activity, as well as its indirect effects via altering the bioavailability of key nutrients such as nitrogen and phosphorus, thereby exerting selective pressure on microbial communities (Wang et al., 2020). In addition to pH, soil nutrients and vegetation parameters showed positive correlations with most diversity indices, indicating that improving soil fertility through rational fertilization can indirectly promote the recovery of microbial diversity during ecological restoration in mining areas. This finding provides empirical support for the “soil–plant–microbe” coordinated restoration theory proposed by Wang et al. (2025).

Structural equation modeling results indicated an extremely significant direct positive effect of fertilizer application on bacterial alpha diversity (*β* = 0.76, *p* < 0.001), which is consistent with findings reported by Wang et al. (2023). In contrast, the direct effects of fertilization on vegetation indices and soil physicochemical properties were not statistically significant, suggesting that microbial community dynamics are primarily regulated through an indirect “vegetation → soil” pathway. This observation implies that the compound microbial inoculant used in this study effectively reshaped bacterial community structure through direct biological mechanisms such as colonization competition, secretion of metabolites, and signal molecule transmission (Li et al., 2021), rather than relying on the conventional soil–vegetation cascade-driven restoration paradigm. Meanwhile, vegetation indices exhibited a strong positive driving effect on soil physicochemical properties, which in turn significantly enhanced bacterial diversity. Together, these results confirm a dual-track restoration mechanism in the alpine mining ecosystem: direct microbial regulation as the primary driver, supplemented by secondary optimization through vegetation–soil interactions. This pattern contrasts with the “soil–plant priority” restoration pathway identified by Zheng et al. (2023) in a mining overburden dump in Inner Mongolia, highlighting the unique nature of microbial response mechanisms in alpine ecosystems.

Principal component score analysis showed that the W3J1 treatment performed best along the PC1 axis (score = 1.16), primarily due to the synergistic enhancement between functional microbes and forage-specific fertilizer within the inoculant–fertilizer co-application system. This finding is highly consistent with Sahur et al. (2025), who reported that the efficacy of functional microbial inoculants depends on their dose-dependent interaction with chemical fertilizers. More importantly, W3J1 achieved the highest overall score, confirming that the optimized ratio (W3J1) effectively drives favorable ecosystem succession by establishing a stable functional microbial community (Huang et al., 2025). These results not only provide a precise dosage optimization guideline for microbial inoculant–fertilizer application in alpine mining areas, but also reveal, from the perspective of microbial niche reconstruction, the underlying mechanisms responsible for differences in restoration performance across treatments.

## 5 Conclusion and perspectives

### 5.1 Conclusion

This study systematically elucidates the synergistic mechanisms of the combined application of forage-specific fertilizer (375.00 kg·hm^−2^) and compound microbial inoculant (350.00 kg·hm^−2^) in the ecological restoration of alpine mining areas. The results show that this specific ratio significantly enhances artificial grassland productivity and improves soil physicochemical properties through the synergistic interaction between functional microbes and the forage-specific fertilizer. From a microbial ecological perspective, the study reveals that the co-application promotes ecosystem recovery via two complementary pathways: direct regulation of bacterial alpha diversity and reconstruction of microbial interaction networks. This finding breaks through the conventional “soil–plant–microbe” restoration framework and establishes a dual-track restoration model in which microbial regulation acts as the primary driver, supplemented by vegetation–soil co-optimization. The study further identifies unique response characteristics in alpine environments, including the coexistence of rapid functional microbial colonization and the maintenance of oligotrophic microbial community stability, along with a distinct fertilization threshold effect. These findings provide both theoretical foundations and technical support for ecological restoration in alpine mining regions. Notably, microbial functions were predicted from 16S rRNA data, which reflects potential rather than actual metabolic activity. Future validation using metagenomic and transcriptomic approaches is needed to confirm functional dynamics.

### 5.2 Perspectives

Future research should deepen understanding of microbial regulatory mechanisms, particularly the molecular signaling pathways mediating interactions between core functional taxa and plant roots. Emphasis should be placed on gene expression networks and their responses to environmental drivers to inform the design of next-generation high-efficiency microbial inoculants. To overcome the limitations of 16S rRNA-based functional prediction, multi-omics approaches especially shotgun metagenomics and metatranscriptomics should be integrated to directly characterize the functional potential and transcriptional activity of soil microbiomes. Long-term field monitoring is essential to assess ecosystem stability and successional dynamics under varying restoration strategies. Combining multi-omics data with artificial intelligence can enable dynamic predictive modeling of restoration outcomes. Moreover, multi-scale validation and strengthened regional demonstration networks are needed to optimize technical parameters and accelerate the translation of research into practical engineering applications. These efforts will refine the theoretical framework of ecological restoration in extreme environments, enhance the precision and scalability of restoration technologies, and support ecological recovery and sustainable development in alpine mining areas.

## Data Availability

The raw microbial sequencing data generated in this study have been deposited in the China National GeneBank DataBase (CNGBdb) under the accession number CNP0008273.

## Footnotes

1
https://cloud.majorbio.com
