# Supplementary material for: Optimized fertilizer–microbe ratios enhance synergistic restoration of alpine mining ecosystems
Source: Front Microbiol. 2025 Dec 12;16:1709528. doi: 10.3389/fmicb.2025.1709528 (PMC12742312; doi:10.3389/fmicb.2025.1709528)
Supplement: Supplementary file 1 [file Data_Sheet_1.docx]

**Figure S1.** Geographic location and meteorological data of the experimental site.

**Figure S2.** Schematic illustration of vegetation growth dynamics at the experimental site.

**Figure S3.** LEfSe analysis of soil bacterial communities under different treatments.

**Figure S4.** Changes in topological features of soil bacterial single-factor correlation networks under different treatments.

**Figure S5.** Single-factor correlation network analysis of soil bacterial communities under different treatments.

**Figure S6.** Hierarchical clustering analysis of soil bacterial communities under different treatments.


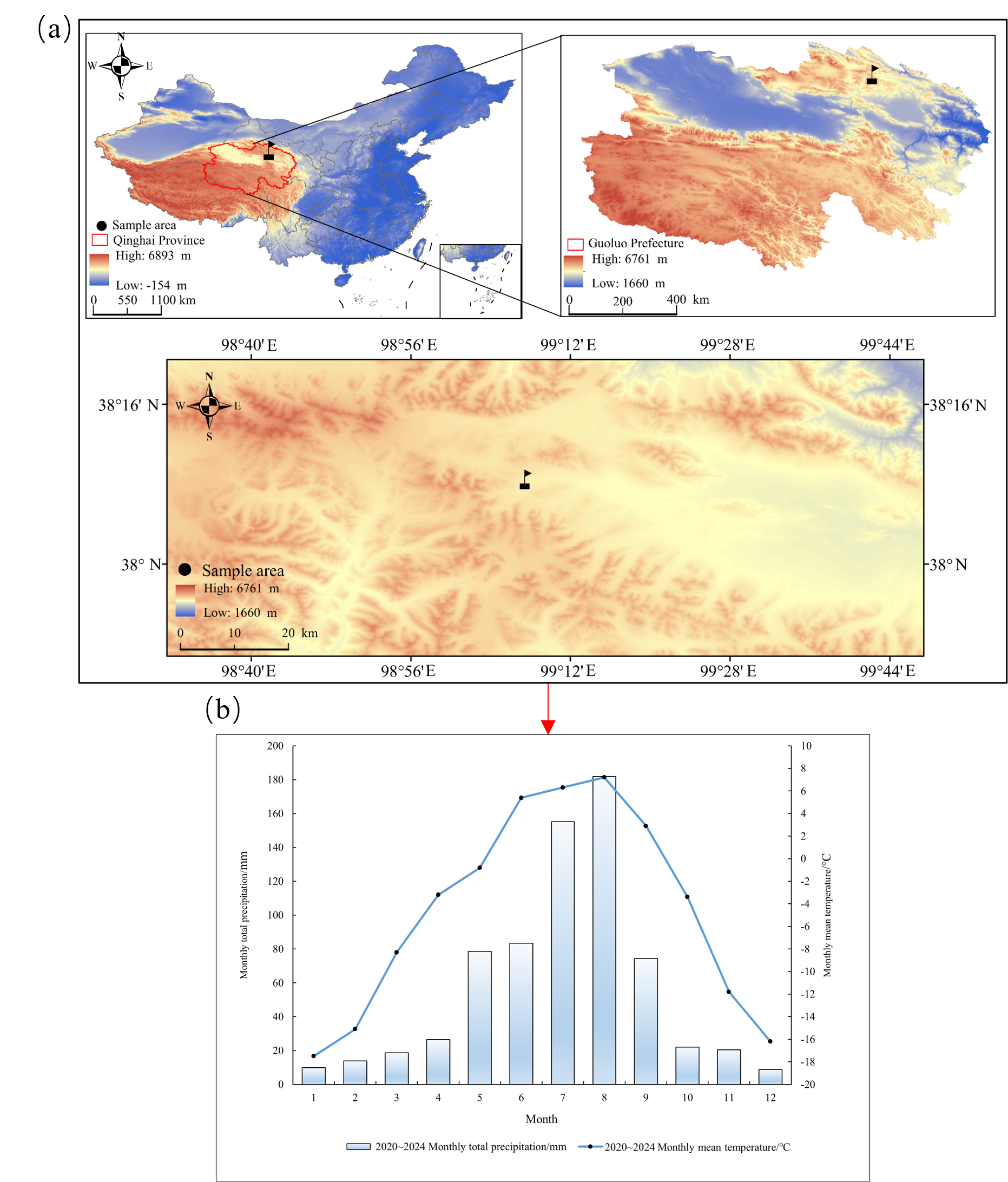


**Note**: (a) Geographic location of the experimental site; (b) Monthly mean temperature and monthly total precipitation at the experimental site from 2020 to 2024 (meteorological data sourced from the Tianjun County National Meteorological Station).
**Figure S1.** Geographic location and meteorological data of the experimental site.


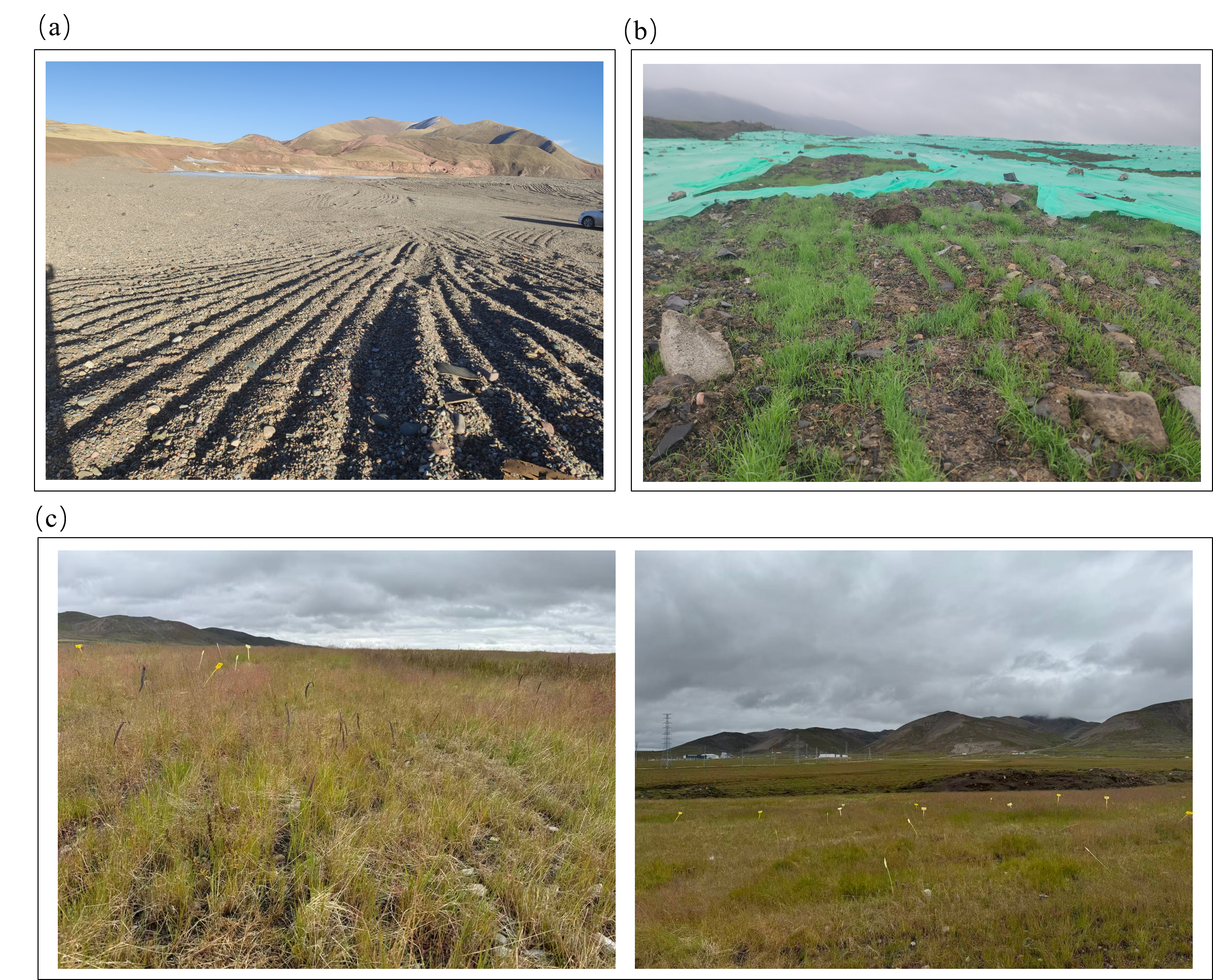


**Note**: (a) Initial stage of the experiment before planting; (b) seedling emergence 45 days after planting; (c) vegetation community landscape after two consecutive years of fertilization treatments. The experimental period spanned from 2022 to 2024.

**Figure S2.** Schematic illustration of vegetation growth dynamics at the experimental site.


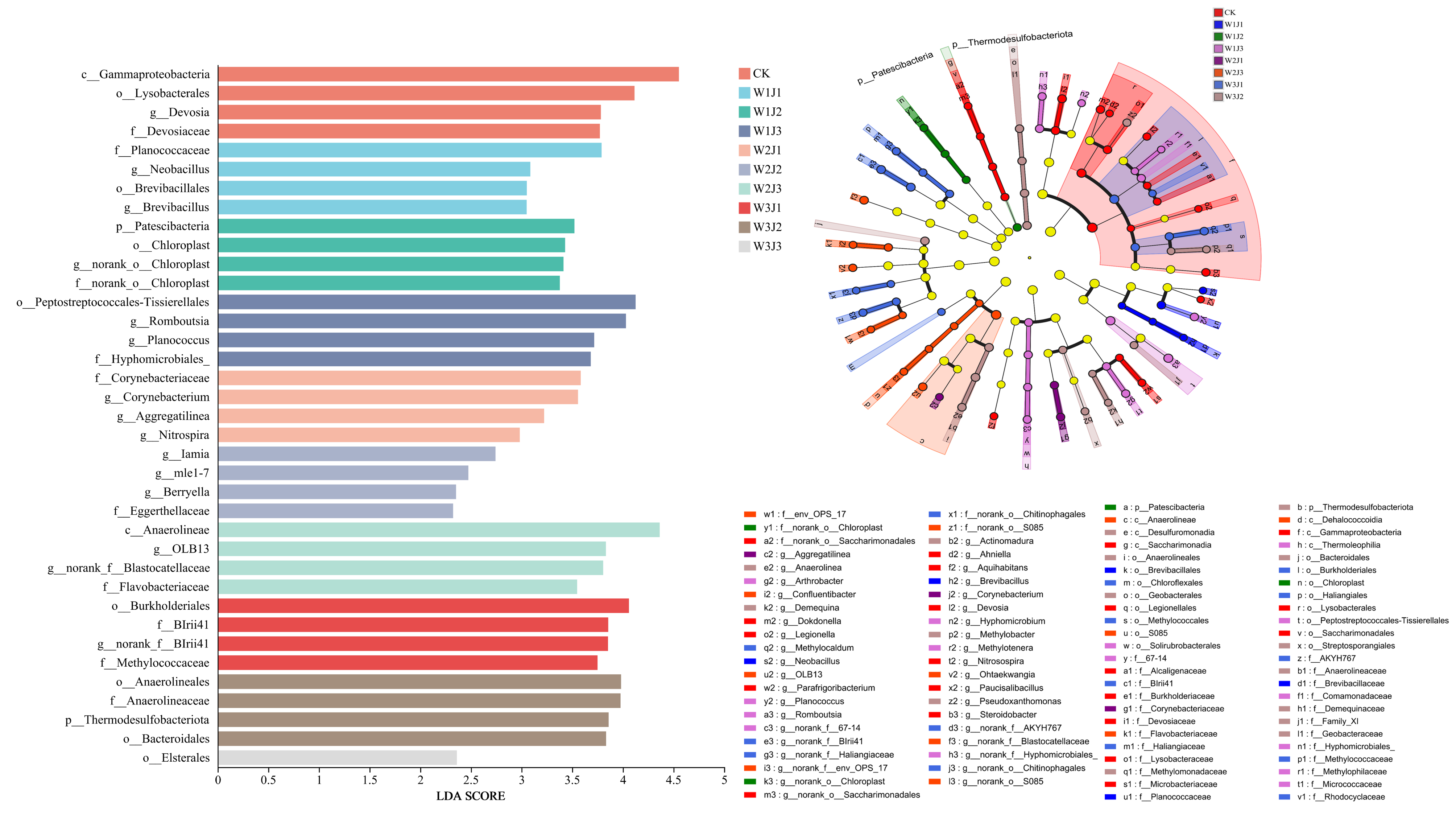


**Note**: W represents the application level of forage-specific fertilizer (W1: 225.00 kg·hm⁻²; W2: 300.00 kg·hm⁻²; W3: 375.00 kg·hm⁻²); J represents the application level of compound microbial inoculant (J1: 350.00 kg·hm⁻²; J2: 500.00 kg·hm⁻²; J3: 650.00 kg·hm⁻²).

**Figure S3.** LEfSe analysis of soil bacterial communities under different treatments.


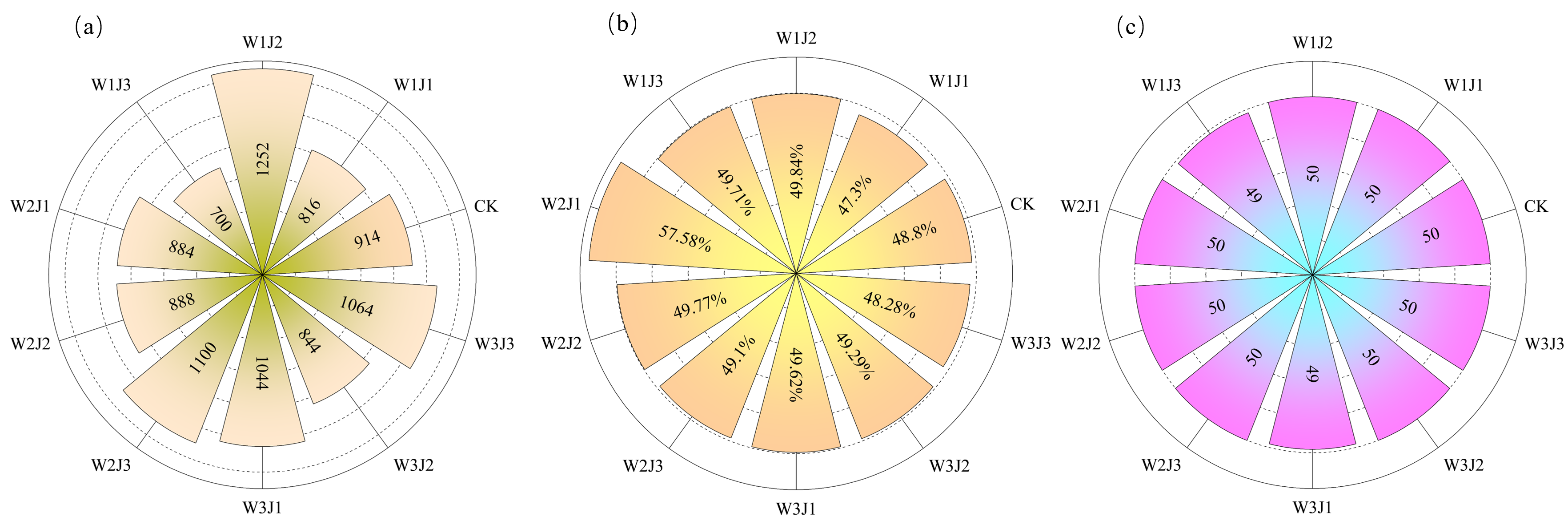


**Note**: Figure 6a shows the number of network edges; Figure 6b shows the proportion of positive correlations; Figure 6c shows the number of network nodes. W represents the application level of forage-specific fertilizer (W1: 225.00 kg·hm⁻²; W2: 300.00 kg·hm⁻²; W3: 375.00 kg·hm⁻²); J represents the application level of compound microbial inoculant (J1: 350.00 kg·hm⁻²; J2: 500.00 kg·hm⁻²; J3: 650.00 kg·hm⁻²).

**Figure S4.** Changes in topological features of soil bacterial single-factor correlation networks under different treatments.


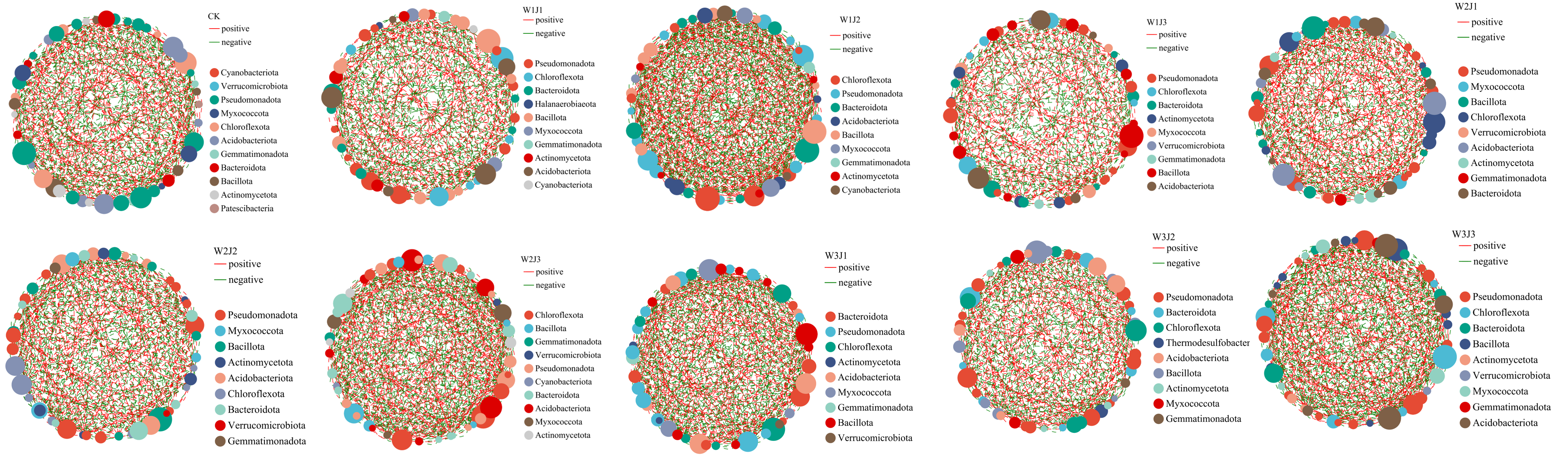


**Note**: W represents the application level of forage-specific fertilizer (W1: 225.00 kg·hm⁻²; W2: 300.00 kg·hm⁻²; W3: 375.00 kg·hm⁻²); J represents the application level of compound microbial inoculant (J1: 350.00 kg·hm⁻²; J2: 500.00 kg·hm⁻²; J3: 650.00 kg·hm⁻²).

**Figure S5.** Single-factor correlation network analysis of soil bacterial communities under different treatments.


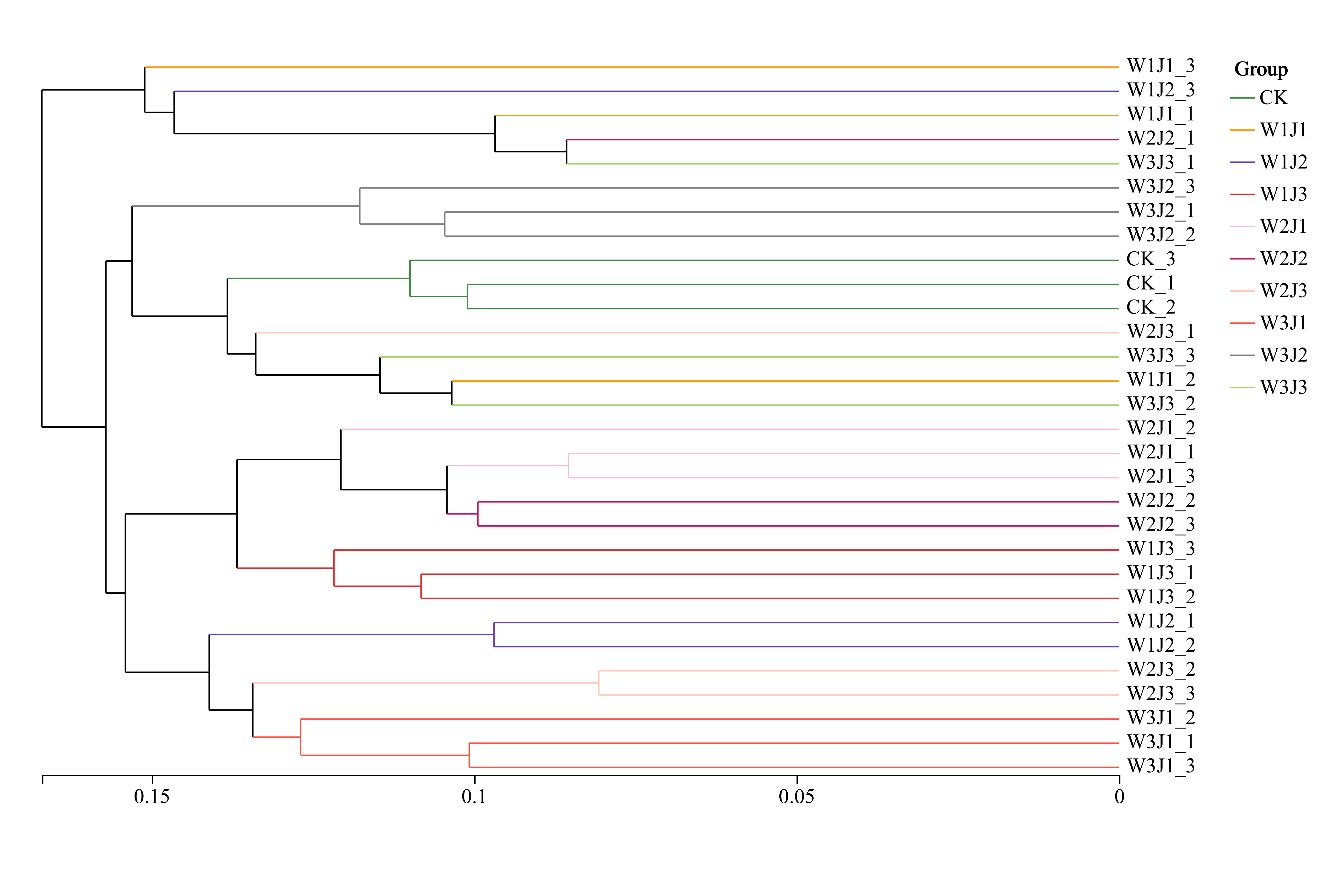


**Note**: W represents the application level of forage-specific fertilizer (W1: 225.00 kg·hm⁻²; W2: 300.00 kg·hm⁻²; W3: 375.00 kg·hm⁻²); J represents the application level of compound microbial inoculant (J1: 350.00 kg·hm⁻²; J2: 500.00 kg·hm⁻²; J3: 650.00 kg·hm⁻²).

**Figure S6.** Hierarchical clustering analysis of soil bacterial communities under different treatments.
